# The Extracellular Matrix Component Psl Provides Fast-Acting Antibiotic Defense in *Pseudomonas aeruginosa* Biofilms

**DOI:** 10.1371/journal.ppat.1003526

**Published:** 2013-08-08

**Authors:** Nicole Billings, Maria Ramirez Millan, Marina Caldara, Roberto Rusconi, Yekaterina Tarasova, Roman Stocker, Katharina Ribbeck

**Affiliations:** 1 Department of Biological Engineering, Massachusetts Institute of Technology, Cambridge, Massachusetts, United States of America; 2 Ralph M. Parsons Laboratory, Department of Civil and Environmental Engineering, Massachusetts Institute of Technology, Cambridge, Massachusetts, United States of America; University of Washington, United States of America

## Abstract

Bacteria within biofilms secrete and surround themselves with an extracellular matrix, which serves as a first line of defense against antibiotic attack. Polysaccharides constitute major elements of the biofilm matrix and are implied in surface adhesion and biofilm organization, but their contributions to the resistance properties of biofilms remain largely elusive. Using a combination of static and continuous-flow biofilm experiments we show that Psl, one major polysaccharide in the *Pseudomonas aeruginosa* biofilm matrix, provides a generic first line of defense toward antibiotics with diverse biochemical properties during the initial stages of biofilm development. Furthermore, we show with mixed-strain experiments that antibiotic-sensitive “non-producing” cells lacking Psl can gain tolerance by integrating into Psl-containing biofilms. However, non-producers dilute the protective capacity of the matrix and hence, excessive incorporation can result in the collapse of resistance of the entire community. Our data also reveal that Psl mediated protection is extendible to *E. coli* and *S. aureus* in co-culture biofilms. Together, our study shows that Psl represents a critical first bottleneck to the antibiotic attack of a biofilm community early in biofilm development.

## Author Summary

Many bacteria have the ability to form multicellular communities, termed biofilms. An important characteristic of a biofilm is the ability of cells to synthesize and secrete an extracellular matrix. This matrix offers structural support, community organization, and added protection, often making the cells impervious to desiccation, predation, and antimicrobials. In this study, we investigate the contributions of polysaccharide components found in the extracellular matrix of *Pseudomonas aeruginosa* at progressive stages in biofilm development. We first show that one specific polysaccharide, Psl, provides an added defense for *P. aeruginosa* biofilms against antimicrobials of different properties for young biofilms. Then, by cultivating biofilms that contain both Psl producing and Psl non-producing strains, we find that *P. aeruginosa*, *E. coli*, and *S. aureus* species that lack Psl take advantage of the protection offered by cells producing Psl. Collectively, the data indicate that Psl is likely to play a key protective role in early development of *P. aeruginosa* biofilm associated infections.

## Introduction

Hydrogels have broad applications in nature and form the basis of vital selective barriers such as mucus, the tissue extracellular matrix, and nuclear pores [1]. One important hydrogel barrier is found in the extracellular matrix of bacterial biofilms [2]–[4]. The biofilm matrix is secreted by, and surrounds, bacteria within a biofilm. It confers adhesion to substrates and between the cells [5], [6], but it also serves as a selective filter, allowing the entry of nutrients [2], [7] while delaying passage of certain antimicrobials [8]–[10]. The biofilm matrix is essential for bacterial defense against environmental insults, yet the components and mechanisms that govern its selectivity for small molecules, such as nutrients, toxins, or antimicrobials, are still largely unknown.

The biofilm matrix is composed of diverse macromolecules including proteins, extracellular DNA, and lipids. In addition, like many other hydrogel barriers [11]–[15], the biofilm matrix contains different types of polysaccharides. The biological function of sugars outside metabolism is poorly understood: controlling the filtration properties of hydrogels may be one of their central functions. Indeed, alterations in polysaccharide composition and concentration correlate with biofilm development. During initial stages of biofilm formation, exopolysaccharides facilitate surface and cell-to-cell attachment. As the biofilm matures, exopolysaccharide production increases and diversifies, and contributes to the generation of microcolony formation and more complex architecture [16]. Alterations in polysaccharide composition also contribute to changes in biofilm antibiotic resistance [17], [18]. Overall, the presence of a biofilm matrix, can lead to increased resistance to antimicrobials and the host immune system simply not observed in their free-swimming counterparts [2]. As a result, biofilms can cause particularly devastating chronic infections or facilitate life-threatening nosocomial infections in short time courses [19]–[24]. A biofilm's resilience to eradication can also cause significant damage in environmental and industrial settings, such as on ship hulls [25] and water pipeline systems [26].

Here, we investigate the role of individual polysaccharides on the permeability of *Pseudomonas aeruginosa* biofilm matrix to antibiotics. The gram-negative bacterium *P. aeruginosa* is an avid biofilm former that is implicated in both chronic and acute infections [27]. It represents an ideal model system to unravel the barrier function of the biofilm matrix, because several components of its matrix have been identified and partly characterized [18], [28]–[33]. In addition, clinical and environmental isolates with varying compositions of exopolysaccharides are available, allowing a direct comparison between extracellular defenses evolved in nature and those formed by synthetically derived laboratory strains [34]–[38]. *P. aeruginosa* produces three major exopolysaccharides found within the matrix: alginate, Pel, and Psl. In the laboratory strains *WT* PAO1 and *WT* PA14, alginate is not a critical matrix component [28]. However, alginate overproduction is a characteristic of mucoid clinical isolates found in the cystic fibrosis lung [39], [40]. Alginate is comprised of blocks of β-1,4-linked d-mannuronic acid residues and its 5-epimer l-guluronic acid [41], [42]. Pel, a glucose rich exopolysaccharide, is important for air-liquid interface pellicle formation [31], [32] and provides a structural scaffold during micro- and macro-colony formation in *WT* PAO1 biofilms [18], [43]. The charge-neutral exopolysaccharide Psl is comprised of D-mannose, D-glucose, and L-rhamnose arranged in pentasaccharide repeats and provides structural support during biofilm formation, playing a role in both cell to cell and cell to substrate attachment [29], [30], [43].

To dissect the contributions of individual polysaccharides to the matrix barrier at selected time points, we use antibiotic tolerance as a reporter. Clinically relevant antibiotics with different charges and mechanisms of action were selected for this study. By comparing the efficacy of antibiotics against biofilms formed by strains that lack different matrix components, we can assess the importance of each polysaccharide in providing tolerance to a specific antibiotic. We found in both static and continuous-flow biofilm experiments, that genetic depletion of Psl result in sensitization toward a range of antibiotics for young biofilms, suggesting that Psl is a critical determinant for the resistance properties of the biofilm matrix at initial developmental stages. We also show that cells devoid of Psl (*P. aeruginosa* Δ*psl*, *S. aureus*, and *E. coli*) can co-exist with Psl-containing biofilms and effectively increase their tolerance. We speculate that Psl can inhibit the function of a range of charged antibiotics by sequestering them, and that removal of Psl in a clinical setting would greatly enhance the efficacy of antibiotic treatments for early onset infections.

## Results and Discussion

To dissect the contribution of individual polysaccharides to the matrix barrier function we first tested their role in tolerance toward the antibiotic colistin, a critical last-resort antibiotic for multidrug resistant *P. aeruginosa* [44], [45]. Colistin belongs to the family of polymyxin cationic antimicrobial peptides, which acts by disrupting the cell membrane [44]. Since it is critical to address infections at initial onset, particularly in burn and wound cases, we examined the contribution of polysaccharide components at early stages of biofilm development. [10], [18]. One important part of our protocol is to examine the killing effect of colistin upon short exposure (2-hour). This exposure period is significantly shorter than standard over-night and 24-hour treatments [18], [46], [47] and approximates the time an antibiotic is available during a one-time treatment before it is metabolized or digested [48]. This is in contrast to other studies that analyze the roles of *P. aeruginosa* exopolysaccharides toward antibiotic tolerance over longer exposure times in more mature biofilms [10], [18].

**Figure 1:**
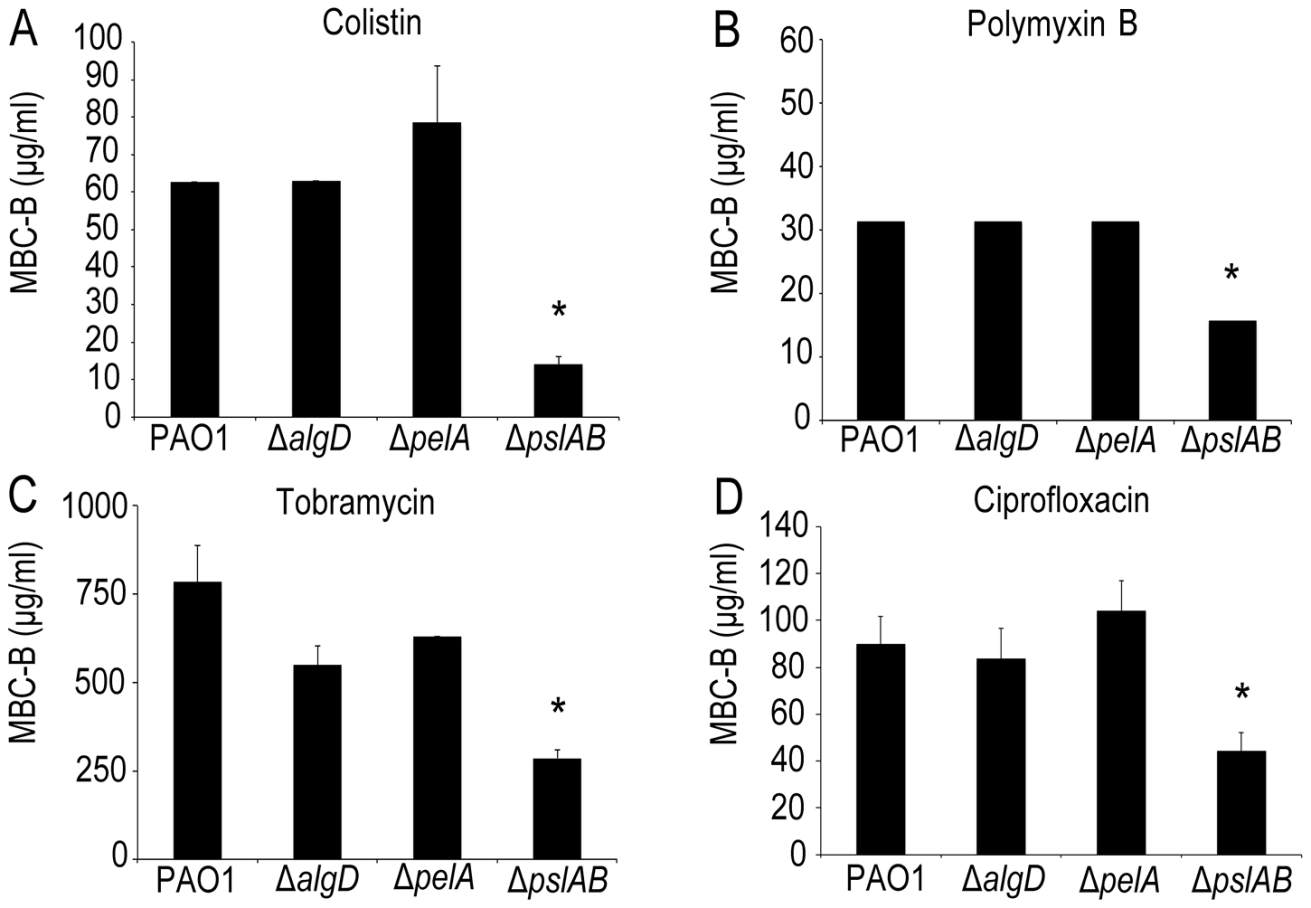
The exopolysaccharide Psl promotes *P.* *aeruginosa* biofilm tolerance to both cationic and anionic antibiotics. Results of the MBC-B assay reveal that removal of Psl increases sensitivity to positively charged colistin (A) and tobramycin (B) and polymyxin B (C). In addition, removal of Psl sensitizes biofilms to negatively charged ciprofloxacin (D). (*) indicates statistical significance from *WT* PAO1 as determined by a student's t-test (P<0.05). Error bars represent SEM (*n* = 3).

Using a microtiter plate assay [31], [49], we determined the minimal colistin concentration required to kill biofilms (the minimal bactericidal concentration for biofilms, MBC-B) formed by wild type PAO1 (*WT*). Experiments were repeated for strains lacking expression of either of the three identified *P. aeruginosa* exopolysaccharides, alginate (*ΔalgD*), pel (*ΔpelA*), and psl (*ΔpslAB*). Fig. 1A shows that 63 µg/ml colistin were needed to eradicate *WT* PAO1 biofilms, whereas only 15 µg/ml were required to eradicate biofilms lacking Psl, which was more than a four-fold decrease in MBC-B in the absence of Psl. In contrast, the MBC-B for alginate-free biofilms (*ΔalgD*) and Pel-free biofilms (*Δpel*) were not significantly different from the MBC-B for the wild type. This suggests that Psl, but not Pel or alginate, can form a first line of defense against colistin for short-term antibiotic for 24-hour biofilms. Colistin sensitivity was not altered for cells lacking a functional *algD* gene product. This result is somewhat expected because alginate is not abundantly expressed in *WT* PAO1 *in vitro* laboratory models early in biofilm development [28]. We are therefore cautious in the interpretation of this result. The lack of Pel was previously shown to sensitize 24 to 48-hour biofilms to aminoglycosides in the laboratory strain PA14, but not for *WT* PAO1, consistent with the results presented here [18]. In parallel to the MBC-B assay, which reveals the concentration required to eradicate all cells in biofilm, we also determined the reduction in viable colony forming units (CFUs) before and after exposure to a fixed concentration of antibiotic. Biofilms were exposed to 32 µg/ml colistin for two hours (Figure S1A) and viable CFUs were quantified on agar plates. At this concentration of colistin, *ΔpslAB* cells were eradicated, whereas *WT* PAO1, *ΔalgD*, and *ΔpelA* biofilms were able to persist. This line of experiments confirmed our conclusion that Psl can mediate protection against colistin for 24-hour biofilms.

To examine the contribution of each polysaccharide also in more mature biofilms, we assayed the sensitivity to colistin of biofilms that had grown for 48 and 72 hours. These results show that Psl exerts a protective effect for 24-hour old biofilms, but did not greatly influence biofilm susceptibility after 48 and 72 hours of maturation (Figure S1A). Additionally, these data show that neither *alg* nor *pel* are critical for biofilm tolerance toward colistin at any time point of development tested here (Figure S1A).

**Figure 2:**
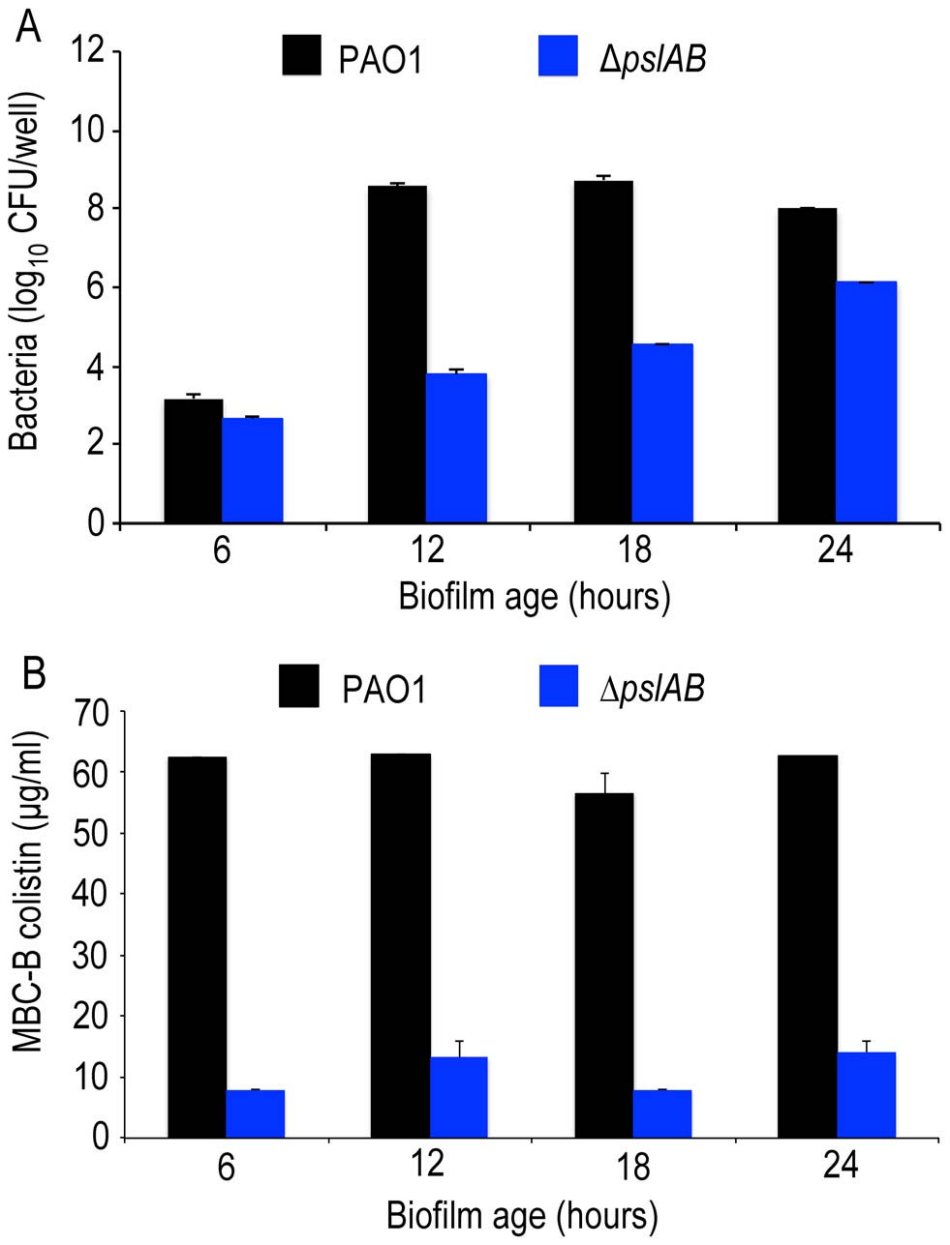
Psl protection is independent of biofilm age up to 24 hours. (A) Cell number count for *WT* PAO1 and Δ*pslAB* at 6, 12, 18, and 24 hours in biofilm development (B) The MBC-B of *WT* PAO1 and Δ*pslAB* at 6, 12, 18, and 24 hours suggest that sensitivity to antibiotics was the result of modulating the matrix and not a consequence of cell number in immature biofilms. Error bars represent SEM (*n* = 3).

*ΔpslAB* cells form biofilms more slowly than wild type cells [30], [50] and have a reduced total biomass compared to *WT* PAO1 (Figure 2A, S3). Hence, to address the possibility that increased colistin sensitivity for *ΔpslAB* biofilms was caused by lower cell numbers, rather than an altered matrix composition, we determined the MBC-B for *WT* and *ΔpslAB* biofilms at multiple time points during early biofilm development. Figure 2B illustrates that the MBC-Bs after 2-hour colistin exposure were independent of biofilm age and, for *WT*, remained constant for 6, 12, 18 and 24-hour biofilms. Together these results suggest that the increased sensitivity to colistin of the *ΔpslAB* biofilms was not due to fewer cells present in the biofilm, but, rather, to the lack of Psl in the biofilm matrix, which in turn appears to affect the interaction of the antibiotic with the cells.

To determine if Psl tolerance against colistin is effective only if cells are within a biofilm, we examined the minimal inhibitory concentration (MIC) of colistin for *WT* and *ΔpslAB* stationary phase planktonic cells normalized to the same cell density. Even in the planktonic state, Psl is constitutively expressed and localizes to the cell surface in *WT* PAO1 [51]. We found a 4-fold reduction in tolerance to colistin for planktonic *ΔpslAB* (Table 1). Although this shift in sensitivity is not as pronounced as in the biofilm state, this data suggests that Psl may contribute to protection for planktonic cells, even in the absence of any protective structure and changes in cellular physiology that arise from the biofilm. Differences in MIC relative to *WT* PAO1 were not observed for planktonic *ΔalgD* or *ΔpelA* (Table 1).

**Table 1:** The minimal inhibitory concentration (MIC) of colistin for stationary phase cells normalized to equal cell density, and the minimum bactericidal concentration of colistin for biofilms (MBC-B) after a 2 hour exposure.

Strain	MIC (colistin µg/ml)	MBC-B (colistin µg/ml)
PAO1	12	63
PA14	12	24
Δ*pslAB*	3	15
Δ*algD*	12	63
Δ*pelA*	12	78
*P*_BAD-_ *psl*	12	125
*P*_BAD-_ *psl* (un-induced)	6	24
CF127	12	125

Is the barrier effect of Psl specific to colistin or does it extend to other clinically relevant antibiotics? To address this question we tested if the loss of Psl would also affect sensitivity toward another cationic antimicrobial peptide, polymyxin B. In addition, we tested sensitivity toward the aminoglycoside tobramycin, a vital first-round treatment of Pseudomonal associated infections [52], [53], and the fluoroquinolone ciprofloxacin, an antibiotic used commonly in *P. aeruginosa* infections due to the ease of oral dosing and limited toxicity. As with colistin, we observed an increase in sensitivity (as determined by the MBC-B) of *ΔpslAB* biofilms relative to *WT* biofilms for polymyxin B (Figure 1B 32 µg/ml *WT* PAO1, 16 µg/ml *ΔpslAB*), tobramycin (Figure 1C; 785 µg/ml *WT* PAO1, 285 µg/ml *ΔpslAB*), and ciprofloxacin (Figure 1D; 90 µg/ml *WT* PAO1, 54 µg/ml *ΔpslAB*). The antibiotic sensitivity was also tested for each antibiotic at different times of biofilm development (24, 48, and 72 hours). As observed before, Psl-mediated protection was critical earlier in biofilm development (24 hours), but dispensable at later time points (S1B; S2A,B). Of note is that *Δpel* and *ΔalgD* biofilms did not show an altered antibiotic sensitivity compared to *WT* biofilms at 24 hours. However, *Δpel* biofilms show a modest decrease in viability at 48 hours when treated with tobramycin or colistin, and also at 72 hours when treated with tobramycin. Together, these results suggest that Psl not only protects cells from colistin, but also can suppress the function of additional antibiotics at initial stages of biofilm development.

**Figure 3:**
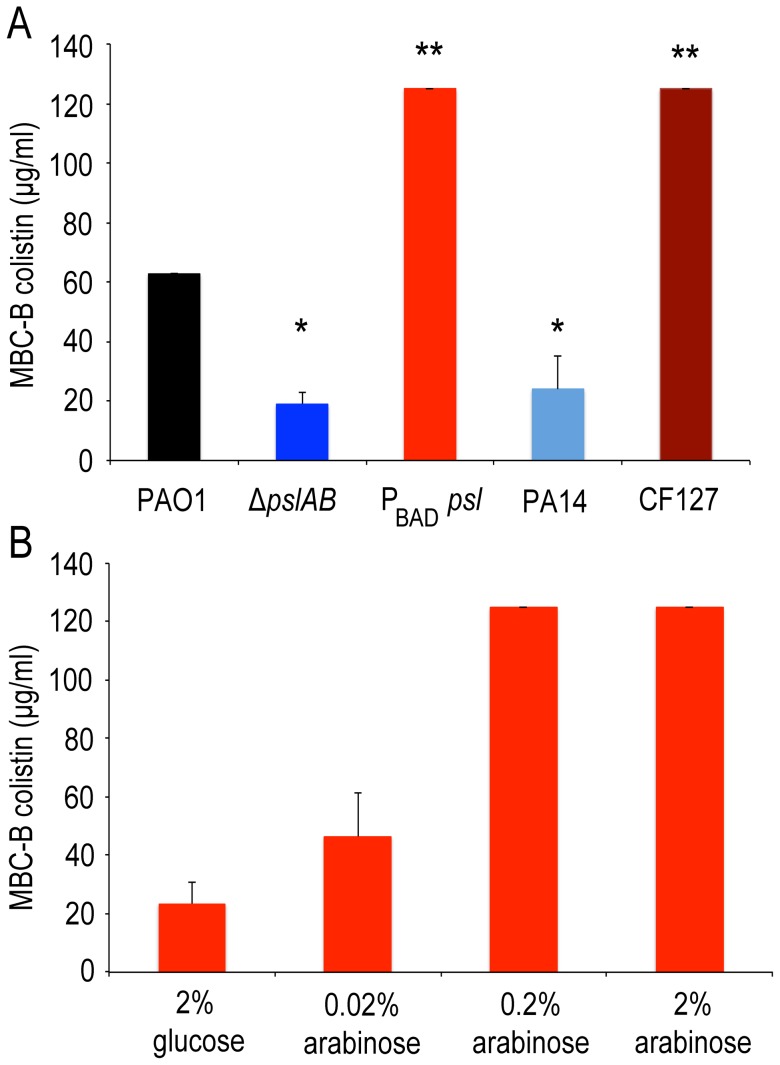
Over-expression of *psl* increases biofilm tolerance to colistin. Opposite to Δ*pslAB*, Psl over-producing strain *P*_BAD-_ *psl* shows increased tolerance to colistin. (A) Strains that naturally lack (PA14) and over-express Psl (CF127) have similar sensitivities as the synthetic Δ*pslAB* and *P*_BAD-_ *psl*, respectively. (B) Since Psl production was directly controllable in the over-producing strain by modulating the amount of the inducer (arabinose) in the culture medium, we could determine if tolerance correlated to the levels of Psl in the biofilm matrix. Biofilms that were formed in the presence of glucose tightly inhibited the expression of *P*_BAD-_ *psl*, which resulted in increased sensitivity to colistin similar to *ΔpslAB*. For the data presented, shades of blue represent strains that do not produce Psl while shades of red represent strains that over-produce Psl. (*) indicates a significantly lower bactericidal antibiotic concentration and (**) indicates a significantly higher bactericidal antibiotic concentration when compared to *WT* PAO1 as determined by a student's t-test (P<0.05). Error bars represent SEM (*n* = 3).

If a deletion of Psl renders biofilms more sensitive to the antibiotics tested here, then we would expect that elevated levels of Psl have the opposite effect and increase antibiotic tolerance. To test this we used a strain derived from *WT* PAO1 where the native *psl* promoter was replaced with an arabinose-inducible promoter (*P*_BAD-_ *psl*). We found that colistin tolerance directly correlates with the levels of Psl produced, rising from an MBC-B of 24 µg/ml to 125 µg/ml in dependence of the level of Psl overexpression (Figure 3A, B). This result was confirmed for colistin, polymyxin B, and tobramycin with antibiotic sensitivity assays (S1A, B; S2A) at 24 hours.

We also compared the tolerance of the synthetically derived *ΔpslAB* to a strain that naturally lacks Psl (PA14). PA14 does not produce Psl owing to a 3-gene deletion in the *psl* operon [32]. The lack of Psl in the PA14 matrix was confirmed by staining of the biofilms with fluorescently labeled HHA lectin, which binds to Psl [54] (Figure S4). 24-hour PA14 biofilms were with a MBC-B of 24 µg/ml similarly sensitive to colistin as *ΔpslAB* (Figure 3A). This result was supported with viability counts for cells exposed to colistin and polymyxin B (Figure S1A, B). Notably, more mature PA14 biofilms at 48 and 72 hours had developed an increased tolerance to colistin and polymyxin B, similar to *ΔpslAB* at these later time points (Figure S1A, B). However, in contrast to *ΔpslAB* biofilms, PA14 biofilms at 24 hours were more tolerant to the aminoglycoside tobramycin. This is in agreement with a previous report, which demonstrated that the Pel rich matrix of PA14 provides protection against aminoglycoside antibiotics [18].

In the converse experiment we measured colistin tolerance of CF127, a natural isolate that secretes increased levels of Psl compared to *WT* PAO1 [38]. The CF127 biofilm grows in distinct microcolonies (Figure S4), and staining with HHA lectin [54] showed that Psl localizes to the CF127 microcolonies (Figure S4). The MBC-B of CF127 toward colistin was 125 µg/ml and hence, comparable to that of the overproducing *P*_BAD-_ *psl* strain (Figure 3A). Interestingly, the increased colistin tolerance of CF127 compared to *WT* PAO1 was not apparent in viability counts (Figure S1A,B; S2A,B). We speculate that structural differences of CF127 biofilms may result in antibiotic tolerance to a sub-population of cells within these structures, which are not resolved in the viability assay.

**Figure 4:**
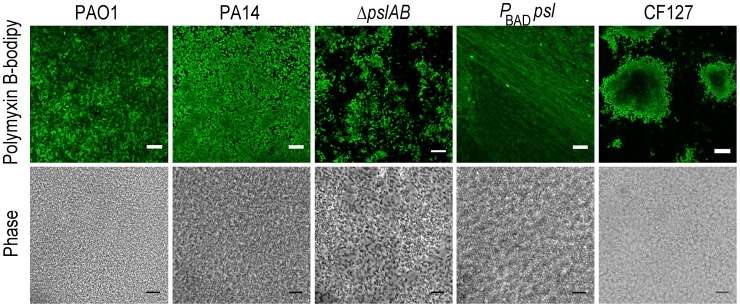
Polymyxin B interacts with the Psl extracellular matrix. Images of *WT* PAO1, PA14, *ΔpslAB*, P_BAD-_ *psl*, and CF127 biofilms subjected to 5 µg/ml fluorescent polymyxin B after 2 hours of exposure. Polymyxin B accumulates in the extracellular material of *P*_BAD-_ *psl* and is less pronounced in *WT* PAO1 biofilms. Fluorescent Polymyxin B did not localize to the matrix in PA14 or *ΔpslAB* biofilms, but instead closely associated with the cell surface. For CF127, Polymyxin B was distributed around the periphery of microcolonies within the biofilm. Scale bars represent 10 µm.

To obtain mechanistic insight into Psl mediated protection, we considered the possibility that Psl may directly sequester antibiotics to the matrix and thereby limit its access to the cell surface. We compared *WT* PAO1, PA14, *ΔpslAB*, P_BAD-_ *psl*, and CF127 biofilms subjected to 5 µg/ml fluorescent polymyxin B after 2 hours of exposure. In the presence of P_BAD-_ *psl* cells the antibiotic distributed along a fibrous matrix heterogeneously throughout the biofilm matrix, and also associated with matrix material in planktonic culture (Figure 4 and S5). The fibrous material was less pronounced for *WT* PAO1, where the localization of polymyxin B was distributed diffusely within the biofilm (Figure 4). This distribution of matrix associated polymyxin B was not observed with the Psl deficient *ΔpslAB* strain or PA14. (Figure 4 and S5). Here, fluorescence was detected in close vicinity of the cell periphery, suggesting that polymyxin B may be interacting with the cell membrane. Of note, polymyxin B localized to the periphery of CF127 microcolonies, but was not observed within the structure.

**Figure 5:**
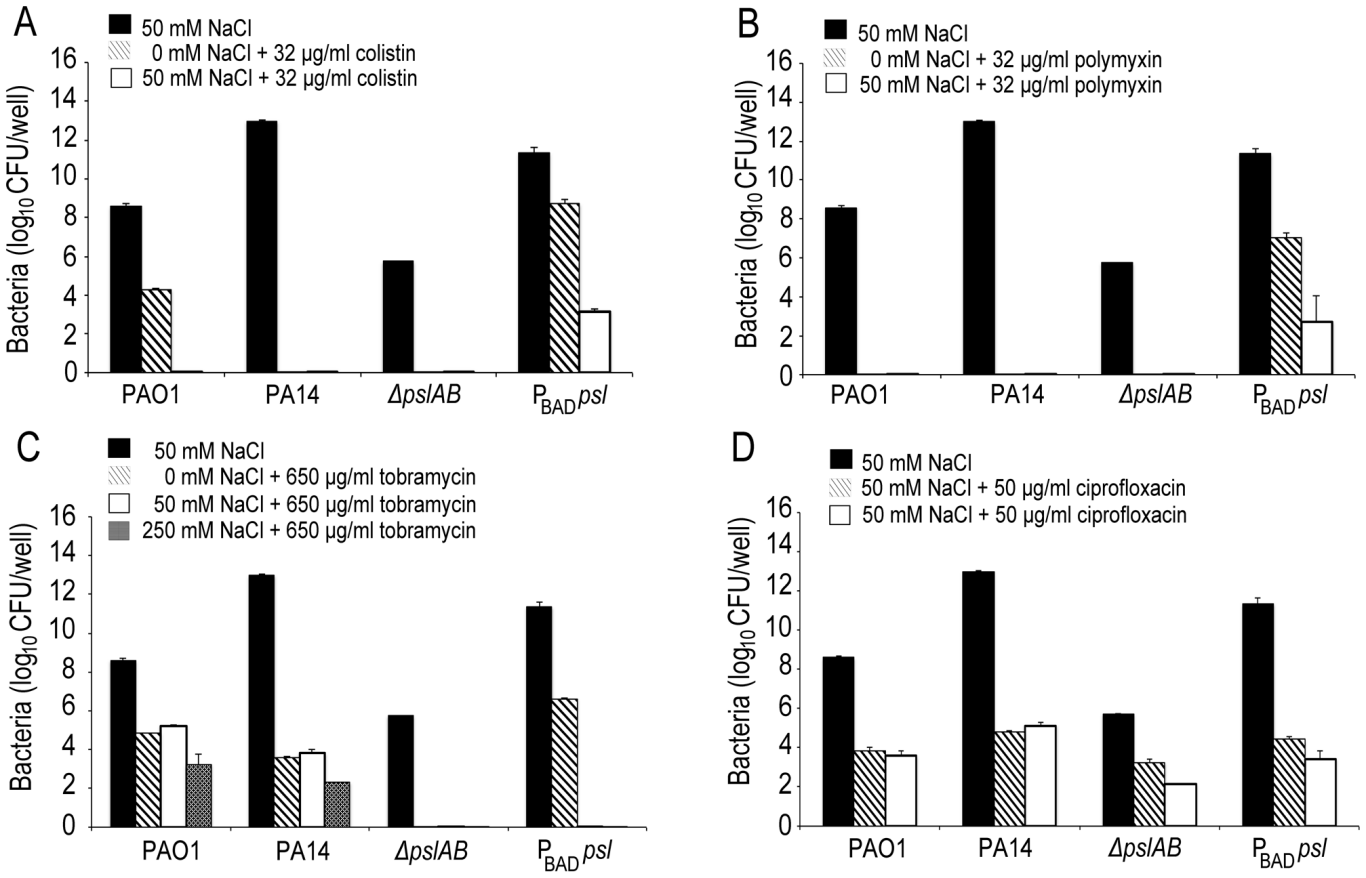
Ionic strength of the challenge medium influences biofilm susceptibility to positively charged antibiotics. Bacterial survival was assessed for *WT* PAO1, PA14, *ΔpslAB*, and *P*_BAD_-*psl* after exposure to colistin (A), polymyxin B (B), tobramycin (C), and ciprofloxacin (D) in the presence or without NaCl. By increasing the ionic strength of the challenge medium with NaCl, electrostatic interactions are predicted to be reduced, leading to an increased efficacy of the positively charged antibiotics colistin, polymyxin B, and tobramycin, but not of ciprofloxacin. Error bars represent SD (*n* = 3).

The binding of fluorescent polymyxin B to the biofilm matrix may, in part, result from electrostatic interactions with the matrix components. To probe for such interactions, we performed antibiotic sensitivity assays at varying ionic strengths through the addition of NaCl to the challenge medium (Figure 5). In growth medium or buffer, charged polymers interact with dissolved ions, which to some extent, form a shell of opposite charges around the molecules. This screening of electrostatic interactions becomes more pronounced with increasing salt concentrations and as a result, the ionic strength in the system will influence the interaction between matrix polymers and diffusing molecules. Specifically, if electrostatic interactions occur between the Psl matrix and the antibiotic molecules, an increase in NaCl concentration may affect these interactions. The challenge medium with 32 µg/ml colistin and no further addition of NaCl reduced the amount of viable cells in a 24-hour *WT* PAO1 biofilm by nearly one half of the total population. However, in the presence of a challenge medium that contained 32 µg/ml colistin and 50 mM NaCl, the total biofilm population was eradicated (Figure 5A). Similar effects were observed for positively charged antibiotics polymyxin B, and tobramycin with a higher concentration of NaCl (250 mM; Figure 5B–C), but not for the negatively charged ciprofloxacin (Figure 5D). We conclude that electrostatic interactions may partly contribute to the sequestration of the antibiotics by the Psl matrix, and that high ionic strength can suppress these interactions, potentially leading to an increased efficacy of the antibiotics. We note that Psl itself is neutrally charged [29], hence, it is conceivable that Psl functions when complexed to other matrix components that could provide the negative charge.

**Figure 6:**
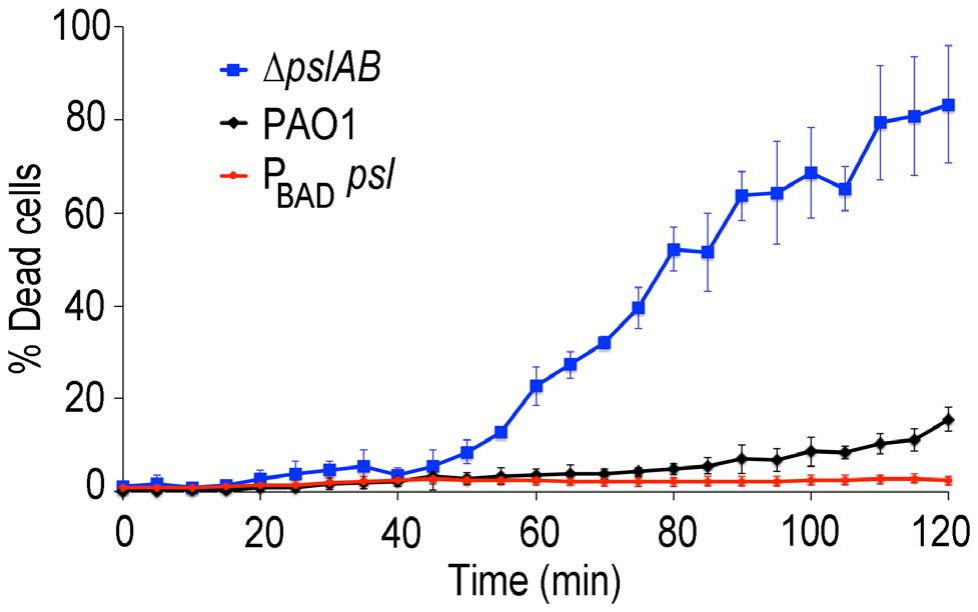
Psl contributes to colistin tolerance for biofilms grown under flow. Colistin kill kinetics for 18-hour old biofilms *WT* PAO1, Psl deficient, and over-producing Psl strains. The Psl deficient strain was substantially more susceptible to colistin compared to the wild type, whereas over-producing Psl was less sensitive. Error bars represent SD (*n* = 3).

In many environments biofilms grow under flow conditions and these may affect the biofilms' barrier properties. To address the role of flow on our findings, we assessed antibiotic susceptibility to colistin in a flow-through microfluidic device. The killing dynamics were examined as biofilms were exposed to 20 µg/ml colistin or buffer without antibiotic for 2 hours (Figure 6; S6). In *WT* biofilms, >80% of the cells survived a 2-hr exposure to the antibiotic (Figure 6 A,B). In contrast, <20% of *ΔpslAB* cells survived, providing further support for our findings and demonstrating that the barrier effect was not compromised by flow and hydrodynamic shear (Figure 6B), although some biomass loss was observed (7% loss for *WT* PAO1 and 12% loss for *ΔpslAB*; Figure S7). Moreover, a biofilm that over-produces Psl (P_BAD-_ *psl*) again shows increased tolerance against colistin compared to a *WT* biofilm (Figure 6B).

**Figure 7:**
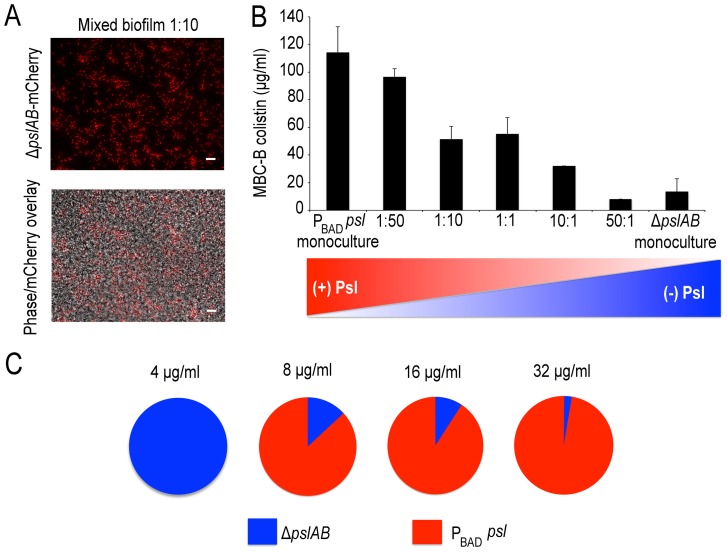
Psl producing cells offer a protective advantage to Psl deficient cells. Mixed *P*_BAD-_ *psl* and *ΔpslAB* (red cells) biofilm population before and after (A) treatment with colistin, cells in the biofilm were removed from the 96 well plate and imaged. (B) MBC-B results for mixed culture biofilms reveal that the presence of *ΔpslAB* sensitized the *P*_BAD-_ *psl* to lower concentrations of colistin. (C) A monoculture of *ΔpslAB* survived exposure to 4 µg/ml of colistin without the requirement of Psl in the matrix. *ΔpslAB* can survive increasing concentrations when part of a joint biofilm with *P*_BAD-_ *psl*, up to 32 µg/ml. Error bars represent SEM (*n* = 3). Scale bars represent 10 µm.

Psl is an extracellular product potentially accessible to foreign cells that are natively devoid of this polymer and hence are, by themselves, more sensitive to antibiotic attack. If non-producing cells are able to coexist with the Psl producers they may be able to exploit the protection by Psl and gain tolerance. This scenario could be relevant in natural settings, where biofilms are often not limited to a single strain or species [55], [56]. We first determined whether *ΔpslAB* cells and the Psl overproducing *P*_BAD-_ *psl* cells could form co-strain biofilms. For this experiment we expressed the fluorescent protein mCherry in *ΔpslAB* cells, mixed them with *P*_BAD-_ *psl* cells to form a co-strain biofilm. Figure 7A shows that *ΔpslAB* cells (red) can indeed grow inside a “Psl donor” biofilm, even if they were incorporated less effectively than the *P*_BAD-_ *psl* cells and therefore represent a smaller proportion of the biofilm. One reason for this is the delay of the *ΔpslAB* cells to attach and mature into biofilms due to their lack of Psl [30] (Figure S3).

The presence of non-producers was not without effect for the entire biofilm, as it weakened the biofilm's tolerance capacity (Figure 7B). We inoculated biofilms with different ratios of *ΔpslAB* and *P*_BAD-_ *psl* cells, and measured the MBC-B for each emerging biofilm. Figure 7B shows that the sensitivity of the composite biofilm toward colistin increased in proportion to the amount of *ΔpslAB* cells present in the initial inoculum. This result suggests that the inclusion of non-producers can reduce the tolerance of the entire biofilm, and that a critical amount of exopolysaccharides per cell is needed for effective protection.

While compromising the overall protective effect from Psl over-producers by becoming part of their biofilm, *ΔpslAB* cells could benefit from the access to the protective exopolysaccharides. We tested if *ΔpslAB* cells within a *P*_BAD-_ *psl* biofilm would survive higher concentrations of colistin than their counterparts growing in a monoculture. Within a monoculture, Δ*pslAB* biofilms could survive colistin concentrations at 4 µg/ml (Figure 7C). In contrast, as part of a joint biofilm with Psl donors, Δ*pslAB* cells were able to survive colistin concentrations up to 32 µg/ml, which would normally kill them (Figure 7C). How many Δ*pslAB* cells the biofilm was able to host without reducing the effective Psl-mediated protection depended on the intensity of the antibiotic attack. By scanning a range of antibiotic concentrations and counting the number of Δ*pslAB* cells that survived treatment, we found that at an antibiotic concentration of 8 µg/ml the biofilm contained 13% Δ*pslAB* cells, while at 32 µg/ml concentration this fraction dropped to 3% (Figure 7C). Thus, Δ*pslAB* cells can benefit from interacting with *P*_BAD_-*psl* cells, even if at the expense of the performance of the Psl-donors. This implies that certain species that lack protective capacity may become more tolerant to therapy as part of mixed-species biofilms.

**Figure 8:**
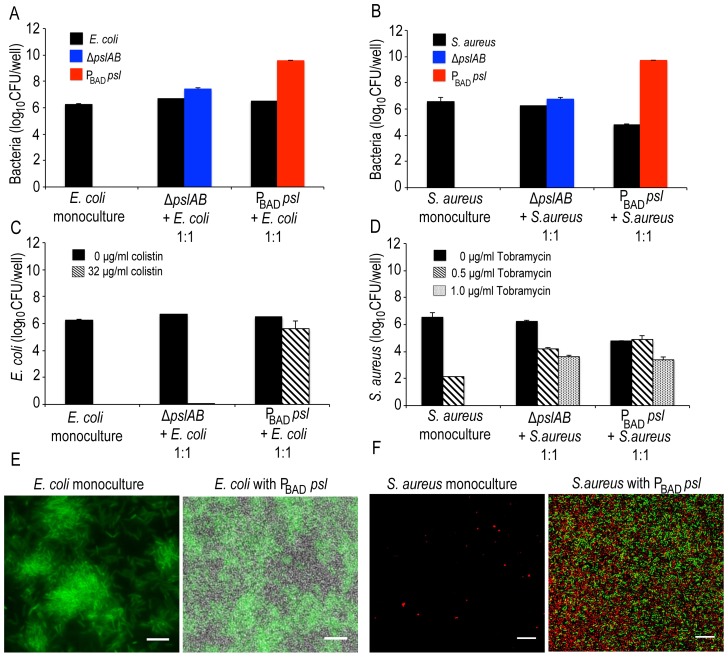
Psl provides a protective advantage for *E.* *coli* and *S. aureus*. Both *E. coli* (A, E) and *S. aureus* (B, F) form mixed species biofilms with *ΔpslAB* and *P*_BAD-_ *psl. E. coli* did not require the presence of *P. aeruginosa* to form a biofilm at the liquid-air interface on polystyrene (E); however, *S. aureus* formed a biofilm at the liquid-air interface only in the presence of *P. aeruginosa* (F). Psl-mediated protection was extendable to *E. coli* biofilms co-cultured with *P*_BAD-_ *psl*. The addition of 32 µg/ml of colistin eradicated monospecies and *ΔpslAB E. coli* mixed species biofilms; however, *E. coli* cells co-cultured with *P*_BAD-_ *psl* were protected (C). Similar effects were observed for *S. aureus*, although a protective effect was also observed with Δ*pslAB* cells (D). *E. coli* cells were identified via expression of GFP while *P. aeruginosa* cells were non-fluorescent (E). *S. aureus* cells were identified with hexidium iodide (red) where as *P. aeruginosa* cells were stained with Syto 9 (green) (F). Scale bars represent 5 µm (E) and 10 µm (F).

Biofilms associated with infections are frequently co-populated by multiple species [57]–[60]. Hence, one important question is if Psl can affect the viability of species that coexist within *Pseudomonas* biofilms. Both gram-negative *E. coli* and gram-positive *Staphylococcus aureus* colonize wounds [61]–[64] and are hence good candidates to address this question. First, we tested if *E. coli* and *S. aureus* form mixed species biofilms when co-cultured with *P*_BAD_-*psl* and Δ*pslAB*, respectively (Figure 8A, B, E, F). *E. coli* readily formed biofilms at the air-liquid interface (Figure 8E) as a monoculture and when co-cultured with *P. aeruginosa*. *S. aureus* formed biofilms at the bottom of a 96 well plate in the absence of *P. aeruginosa*. However, when co-cultured with *P. aeruginosa*, *S. aureus* was incorporated into the air-liquid interface biofilm.

To determine if Psl could provide any advantage for *E. coli*, we quantified *E. coli* sensitivity to 32 µg/ml colistin in the presence and absence of Psl-producing cells. As a monospecies biofilm or when incorporated in a Δ*pslAB* biofilm, *E. coli* was eradicated by this concentration of colistin (Figure 8C). However, when grown together with the *P*_BAD_-*psl*, *E.coli* viability was only mildly compromised by the same treatment (Figure 8C), suggesting that *E. coli* can benefit from the protective effects of *Pseudomonas*-derived Psl. Supporting this result was the MBC-B assay, which shows that the presence of *P*_BAD_-*psl* enhanced tolerance of *E. coli* to 104 µg/ml of colistin (Figure S8A). A similar conclusion might be drawn for *S. aureus*: the monoculture was eradicated with 1 µg/ml of tobramycin and substantially decreased with 0.5 µg/ml, but the cells survived even 1 µg/ml of tobramycin when co-cultured with *Pseudomonas* (Figures 8D). When assessing viable CFUs (Fig. 8D), the protection from the Psl overproducing *P*_BAD_-*psl* strain was only slightly higher compared to the protection from the Δ*pslAB* strain. However, the difference becomes clearer in the MBC-B data (Figure S8B), which shows that *S. aureus* can tolerate a higher concentration of tobramycin in *P*_BAD_-*psl* biofilms than in Δ*pslAB* biofilms, or as monoculture (Figure S8B).

The extracellular matrix in biofilms has long been implicated as a barrier for protection [65], but its exact contribution to resistance is not clear. One reason for this is that the bulk of methods to measure resistance are based on the exposure of cells to antibiotics over long time scales (over-night to 48 hours) [10], [18], [46]. This allows for many cell divisions to occur, giving the cells time to build adaptive mechanisms at the cellular or genetic level. However, these studies may mask any contribution from a physical barrier, which should be apparent at much shorter time scales: if the matrix acts as a true physical shield then matrix-embedded bacteria should show immediate tolerance on exposure to antibiotics.

To focus on the physical barrier effects of the matrix we tested the short-term tolerance response of bacteria, and the contribution of the known matrix polysaccharides within. We found that Psl can provide instant defense and contributes to protecting cells from the action of a broad spectrum of antibiotics with diverse biochemical properties. Psl provides a measure of protection from cationic antimicrobial peptides (colistin, polymyxin B), tobramycin, and to some extent ciprofloxacin. Importantly, this protection is observed in early stages of biofilm development but does not have a profound effect at later time points (48, 72 hour biofilms). As the biofilm continues to develop into the characteristic mushroom shaped microcolonies [18], [30], [43] resulting in spatio-temporial changes in the matrix [43], [51], we conclude that different barrier properties arise from the biofilm structure and other polymers which may be redundant to, or dominate over, Psl function.

Supporting our data on the protective effect of Psl is a recent report that shows that strains producing Psl are capable of growth and biofilm formation in the presence of the anti-biofilm agent Polysorbate 80, a non-ionic surfactant [50]. Psl is found in two forms in the matrix, where large molecular weight oligosaccharide repeats localize around the cell surface [30] and smaller, soluble fractions are distributed throughout the matrix [29]. Based on the localization results of fluorescent polymyxin B, it is possible that the polymer attracts the small antibiotic molecules by direct interaction, as has been proposed for alginate [66], [67] and *ndvB*-encoded periplasmic glucans [10], [68] or reduces affinity of antibiotics to the cell surface. In support of an interaction mechanism, we also show that this attraction may be attributed to, in part, by electrostatic interactions between the antibiotics and the biofilm matrix since the addition of NaCl sensitizes cells with a Psl rich matrix to positively charged antibiotics. Further, the presence of Psl could contribute to indirect effects on antibiotic tolerance such as limiting the diffusion of oxygen or other nutrients, contributing to a more dormant cellular state. However, it is important to note that we did not detect a difference in growth rate for any of the strains. Nevertheless, deciphering the barrier mechanism of Psl may inspire solutions to some vexing treatment challenges in medicine at the initial stages of biofilm associated infections in burns and wounds, where early treatment for bacterial eradication is imperative.

An external barrier as the sole defense mechanism is probably risky, as its capacity to sequester molecules is likely limited. However, such a fast-acting physical barrier may offer cells enough time to build up synergistic and longer-term defense systems. The presence of a physical barrier also implies that it is potentially accessible to more sensitive bacterial species that would otherwise succumb to antibiotic exposure. Our *in vitro* system highlights the possibility that interaction with a protective matrix can render a sensitive strain resistant. Importantly, we observed that Psl mediated protection is extendable to *E. coli* and *S. aureus* which also readily colonize burns and wounds. These results may explain why, in many cases, mixed species biofilms are more tolerant to therapy than their monoculture counterparts [69]–[72]. However, the opposite perspective, where co-habitation of a matrix deficient strain compromises the tolerance properties of the biofilm community as a whole, is also important. From a biochemical standpoint this implies that a certain polymer-to-cell ratio is optimal for protection, and that the polymers can become depleted by excessive amounts of non-producers. From a therapeutic outlook, the depletion of the protective polymers may be considered in future treatment strategies of initial onset infections.

## Materials and Methods

### Strains, culture conditions, and antibiotics

The *Pseudomonas aeruginosa* strains used in this study are as follows: laboratory wild type PAO1, laboratory wild type strain PA14, PAO1Δ*pslAB* (Psl deficient), and PAO1-P*_BAD_psl* (over-producing Psl), PAO1Δ*algD*, PAO1Δ*pelA*, and cystic fibrosis isolate CF127. The mutant strains PAO1Δ*pslAB*, PAO1-P*_BAD_psl*, and PAO1Δ*algD* were a generous gift of Daniel J. Wozniak. PAO1Δ*pel* and cystic fibrosis isolate CF127 were a generous gift of Matthew R. Parsek. Other strains include *E. coli* EMG2 constitutively expressing GFP from pBBR1(MCS5)-P_lac_-*gfp* and *Staphylococcus aureus* UAMS-1 and were used for co-culture experiments. Details and references for all strains can be found in Text S1. All of the *P. aeruginosa* strains and *E. coli* EMG2 were cultured in 1% Tryptone Broth (TB). *S. aureus* was cultured in LB broth for both monoculture and co-culture experiments. Selective agar plates were used to evaluate CFU counts for *P. aeruginosa* (Cetrimide Agar; Sigma-Aldrich 70887) and *S. aureus* (Mannitol Salt Phenol Red Agar; Sigma-Aldrich 63567) co-culture biofilms. Arabinose was maintained culture medium of PAO1-P*_BAD_psl* and in all co-strain/species biofilm experiments at a final concentration of 2% unless otherwise noted. As a control, arabinose was added to the culture medium of *WT* PAO1 to confirm that arabinose did not influence biomass or antibiotic resistance for each. The strain *PAO1*Δ*pslAB* (Psl deficient) was transformed with pMP7605-mCherry [73] (the plasmid construct pMP7605-mCherry was kindly provided by Ellen L. Lagendijk, Institute of Biology, Leiden University, The Netherlands) via standard methods in bacterial conjugation [74]. For *P. aeruginosa* and *E. coli* strains cultures, an OD_600_ of 0.0025 represents a culture density of ∼5.0×10^5^ and for *S. aureus* an OD_600_ of 0.0025 represents a culture density of ∼5.0×10^4^.

Antibiotics from three classes that target *P. aeruginosa* were chosen for investigation (Text S1): polymyxins (colistin sulfate salt Sigma-Aldrich #C4461; polymyxin B sulfate Sigma-Aldrich #P0972), aminoglycosides (Tobramycin Sigma-Aldrich #T4014), fluoroquinolones (Ciprofloxacin Sigma-Aldrich #17850). They were chosen due to their clinical relevance, difference in net charge, and difference in mechanism of action.

### Microtiter biomass assay

The total biofilm biomass for each of the *P. aeruginosa* strains used in this study was quantified with crystal violet staining as previously described [75]. Briefly, biofilms were grown in 96 well polystyrene microtiter plates in 1% TB medium at room temperature for 24, 48 and 72 hours (150 µl of culture diluted to an OD_600_ 0.0025 per well). For 48- and 72-hour biofilms, the medium was aspirated and replaced with fresh 1% TB each day (supplemented with 2% arabinose). At the end of each time point, the medium was aspirated and the plates were washed twice with tap water to remove any planktonic cells. 175 µl of 0.1% crystal violet was added to each well and remained for 10 minutes at room temperature. After staining, the crystal violet solution was aspirated and the plates were washed twice with tap water to remove any residual stain. The plates were allowed to dry for at least 30 minutes, followed by solubilization of the stained biofilm with 175 µl of 33% acetic acid. The resulting absorbance was recorded at 550 nm.

### Lectin staining

HHA-TRITC (EY Labs) was used at a final concentration of 200 µg/ml as previously described [54]. Biofilms were grown at the air-liquid interface on UV sterilized polystyrene surfaces for 24 hours. The biofilms were submerged in the lectin solution for 30 minutes and imaged with a Zeiss LSM 510 Meta Confocal using a 100×/1.4 NA oil immersion objective.

### Minimal Inhibitory Concentration (MIC) assay for stationary phase cells

The MIC was determined by a standard micro-dilution protocol with modifications. Cells grown to stationary phase were normalized to an OD_600_ 0.5 and were exposed to 2-fold series of colistin dilutions in order to determine the minimal concentration of colistin that reduced cell viability within two hours. After the challenge, the planktonic cells were centrifuged at 6000 rpm and washed with PBS to remove residual antibiotic. The cultures for each dilution were plated on LB agar plates without antibiotics to determine the minimum concentration of colistin required to inhibit growth within the two hour time frame.

### Minimal Bactericidal Concentration for Biofilms (MBC-B)

This assay was performed as described previously [49] with modifications. Briefly, mid-exponential phase cultures were normalized to an OD_600_ 0.0025 in 1%TB. 150 µl of diluted culture was added to each well of a polystyrene 96-well microtiter plate and incubated for 24 hours at room temperature. The medium in each well was aspirated to remove planktonic cells. The resulting biofilms were carefully washed with PBS (pH 7.4) to remove any remaining unattached cells. Two-fold dilutions of antibiotics tested were prepared in appropriate solvents and 150 µl of the antibiotic dilutions were added to the biofilm plate (0–1 mg/ml for colistin, 0–1 mg/ml for polymyxin B, 0–10 mg/ml for tobramycin, and 0–1 mg/ml ciprofloxacin). After 2 hours, the antibiotic was removed and the biofilms were carefully rinsed with PBS. 150 µl of PBS was added to each well along with 150 µl of sterile glass beads (Sigma-Aldrich #G8772; 425–600 µm). The plate was covered with sterile aluminum sealing film (Sigma-Aldrich #Z722642) to prevent any cross-contamination between wells. The plate was then vortexed for 5 minutes to remove adherent cells from the polystyrene well. To quantify cell viability, 35 µl per well was plated on LB agar without antibiotics. The lowest antibiotic concentration that inhibited growth was considered to be the minimal bactericidal concentration for the biofilm (MBC-B).

For mixed culture MBC-B analysis, mid-exponential phase cultures were inoculated at different ratios, but the total cell number in solution remained constant when added to each well. To quantify the percentage of Psl deficient survivors after antibiotic challenge, Δ*pslAB* expressing fluorescent mCherry were quantified with phase contrast and fluorescence microscopy using a Zeiss Observer Z.1 epifluorescent microscope with a 40×/0.75 NA dry objective. The percent survival of Psl deficient cells was calculated by determining the number of fluorescent cells relative to the total cell population.

### Antibiotic sensitivity assays

The antibiotic sensitivities of air-liquid interface biofilms on polystyrene 96 well microtiter plates were assessed at 24, 48, and 72 hours. The microtiter wells were inoculated with 150 µl of culture at an OD_600_ of 0.0025. For 48- and 72-hour biofilms, the medium was aspirated and replaced with fresh 1%TB each day. For each time point, the medium was aspirated from the well and gently washed with PBS to removed non-adherent cells. Biofilms were exposed to 32 µg/ml colistin, 32 µg/ml polymyxin B, 650 µg/ml tobramycin, or 50 µg/ml ciprofloxacin for 2 hours. Cells were removed by the glass bead method described above for MBC-B assays. Viability was quantified by serial dilutions and CFU counts of the surviving population.

To evaluate the contribution of electrostatic interactions between matrix components and antibiotics, the antibiotic sensitivity was determined for colistin, polymyxin B, tobramycin, and ciprofloxacin with the addition of 50 mM NaCl. 250 mM NaCl was also evaluated for tobramycin. The effect of NaCl on bacterial attachment was quantified by adding the appropriate concentration of NaCl to the challenge medium without antibiotic. Viability was quantified by serial dilutions and CFU counts of the surviving population.

For determining cell viability of the *P. aeruginosa* mixed culture air-liquid interface biofilms with *E. coli* and *S. aureus*, cultures were inoculated at a 1∶1 ratio. An independent evaluation (CFU counts) of the biofilm population was conducted for each mixed species biofilm to quantify the composition of cells inhabiting the biofilm before antibiotic treatment. For *P. aeruginosa* and *E. coli* mixed biofilms, the ratio of colonies expressing GFP (*E. coli* strain) compared to non-fluorescent cells (*P. aeruginosa*) was determined after plating CFUs. For *P. aeruginosa* and *S. aureus* mixed biofilms, CFU counts for each species were assessed with selective media for each strain.

### Imaging mixed species biofilms

*E. coli* expressing GFP and *P. aeruginosa* strains were inoculated at a 1∶1 ratio (or as monocultures) and grown at the air-liquid interface on UV sterilized polystyrene surfaces for 24 hours. Fluorescence and phase contrast images were acquired to determine the biofilm forming capabilities of *E. coli* at the air-liquid interface on a polystyrene surface both with and without *P. aeruginosa*. A similar procedure was performed for *S. aureus*. To determine the biofilm forming capabilities of *S. aureus* at the air-liquid interface on a polystyrene surface both with and without *P. aeruginosa*, *S. aureus* was stained with the gram-positive specific dye, hexidium iodide (Molecular Probes). *P. aeruginosa* was identified with Syto 9 staining (Molecular Probes).

### Polymyxin B binding assays

Fluorescently labeled Polymyxin B (green-fluorescent BODIPY FL-Polymyxin B; Molecular Probes, Invitrogen) was used at a final concentration of 5 µg/ml. Stationary phase cultures were challenged with 5 µg/ml of Bodipy-polymyxin B for 2 hours. An aliquot of each culture was immobilized on a 1% agarose covered glass slide. Air-liquid interface biofilms grown on UV sterilized polystyrene squares were treated with 5 µg/ml Bodipy-polymyxin B for 2 hours. All images for Bodipy-polymyxin B assays were acquired with a Zeiss LSM 510 Meta Confocal using a 100×/1.4 NA oil immersion objective.

### Microfluidic-based time-kill kinetic assay

A PDMS (Polydimethylsiloxane; Sylgard 184; Dow Corning, MI, USA) microfluidic device was molded from a silicon master yielding a negative imprint of 10 straight microchannels, 100 µm deep/500 µm wide and then bonded to a glass slide. The device was placed on an inverted Nikon TE2000-E (Nikon Instruments, Japan) equipped with an Andor iXon-885 and a 40× long working distance objective for the duration of the experiment. A bacterial suspension (OD_600_ 0.0025) was introduced into the microchannels under continuous flow driven by a syringe pump (PHD Ultra, Harvard Apparatus, MA, USA) at a flow rate of 0.5 µl/min for 18 hours. The biofilms were stained with Bacterial Viability Kit, (Molecular Probes, Invitrogen Inc., Eugene, OR). Colistin, at a final concentration of 20 µg/ml, was introduced into each channel for 2 hours and one untreated channel served as a control. Phase contrast, green and red fluorescence images were recorded for the same field of view every 5 minutes. The cells absorbed propidium iodide after cell death resulting from colistin exposure. Propidium iodide resulted in fluorescence quenching of Syto 9, the green fluorescent dye used to identify living cells. As cell death progressed over time, there was a decrease in green fluorescence due to a quenching effect and not a consequence of cell detachment. The coverage of dead cells in the biofilm was calculated in ImageJ [76] by adjusting the threshold of 8 bit binary images and measuring the area coverage. This data was expressed as a percentage of the total biofilm area (phase-contrast images) for each time point. A Zeiss 510 confocal laser-scanning microscope (CLSM) was used to acquire xyz optical section images before and after colistin treatment of biofilms within the microfluidic device to quantify the amount of biomass loss during treatment.

## Supporting Information

Figure S1 **Psl protects 24-hour biofilms from cationic antimicrobial peptides.** WT PAO1, WT PA14, *ΔpslAB*, *ΔpelA*, *ΔalgD*, *P*_BAD-_ *psl*, and CF127 biofilms were grown for 24, 48, 72 hours in microtiter plates. After a 2-hour treatment with 32 µg/ml of colistin (A) or polymyxin B (B), cell viability was measure and reported at CFU (log_10_). Psl-mediated protection was apparent for 24-hour biofilms, but dispensable at later time points.
(TIF)

Figure S2 **Psl protects 24-hour biofilms from cationic antimicrobial peptides.** *WT* PAO1, *WT* PA14, *ΔpslAB*, *ΔpelA*, *ΔalgD*, *P*_BAD-_ *psl*, and CF127 biofilms were grown for 24, 48, 72 hours in microtiter plates. After a 2-hour treatment with 650 µg/ml of tobramycin (A) or 50 µg/ml ciprofloxacin (B), cell viability was measure and reported at CFU (log_10_). Psl-mediated protection was critical for *ΔpslAB* 24-hour biofilms treated with tobramycin, but was not required at 48 or 72 hours. Tolerance to ciprofloxacin in strains lacking Psl was not as apparent with this assay.
(TIF)

Figure S3 **A deletion of** ***pslAB*** **in the WT PAO1 background reduces total biomass in an** ***in vitro*** **biofilm model.** Crystal violet assays were used to quantify the total biomass for air-liquid interface biofilms grown in 96 well plates for 24, 48, and 72-hour biofilms. The total biomass for *ΔpslAB* was reduced relative to *WT* PAO1 for all time points measured. Although PA14 does not produce Psl, a reduction in biomass was not observed, presumably due to other contributing polymers in the biofilm matrix. Deletions in *pelA or ΔalgD* did not reduce the total biomass relative to *WT* PAO1. Both *P*_BAD-_ *psl* and CF127 had a greater than 2-fold increase in biomass for each time point compared to *WT* PAO1.
(TIF)

Figure S4 **Lectin staining reveals patterns of Psl distribution in 24-hour biofilms.** Fluorescently labeled HHA stained Psl in *WT* PAO1, *ΔpelA*, *ΔalgD*, *P*_BAD-_ *psl*, and CF127 biofilms [54]. Both PA14 and *ΔpslAB* lack Psl in the matrix and did not bind HHA. Psl was distributed as a localized, fibrous material associated with *WT* PAO1 and *P*_BAD-_ *psl* biofilms, while in *ΔpelA* and *ΔalgD*, the Psl matrix was uniformly distributed throughout the biofilm. HHA localized to microcolonies in CF127 biofilms indicating that Psl was enriched in these structures. Scale bars represent 10 µm.
(TIF)

Figure S5 **Polymyxin B interaction with the extracellular matrix in planktonic cells.** Images of over-producing Psl (*P*_BAD-_ *psl*) and Psl deficient (*ΔpslAB*) cells after a 2-hour challenge with fluorescent polymyxin B. Polymyxin B accumulates in the EPS of Psl over-expressing cells, but appears to bind directly to the cell surface in the Psl deletion strain. Scale bars represent 10 µm.
(TIF)

Figure S6 **Biofilms treated with water only did not contribute to cell death in microfluidic channel.** To serve as a control, biofilms grown in microfluidic channels were treated with sterile water only and monitored for cell death for 2 hours. Images of *WT* PAO1 and mutant strain Δ*pslAB* stained with Syto 9 (live cells) and propidium iodide (dead cells) were acquired and compared after 1 hour of treatment with water only.
(TIF)

Figure S7 **Biomass before and after treatment with colistin.** 3-D projection of confocal images of *WT* PAO1 and mutant strain Δ*pslAB* were acquired and compared before (at 24 hours) and after treatment (at 26 hours) with 20 µg/ml colistin. Cells were stained with Syto9 (Molecular Probes) and counted in the series of xyz images. Scale bars represent 25 µm.
(TIF)

Figure S8 **Psl increases MBC-B for** ***E. coli*** **and** ***S. aureus.*** MBC-B assay reveals an increase in tolerance toward colistin for *E. coli* and *P*_BAD-_ *psl* biofilms (A). Tolerance is also observed toward tobramycin for *S. aureus* and *P*_BAD-_ *psl* biofilms, but to a lesser extent (B).
(TIF)

Text S1 **Description of the strains and antibiotics used in this study.**
(DOCX)

## References

[1] Lieleg O, Ribbeck K (2011) Biological hydrogels as selective diffusion barriers. Trends in cell biology 21: 543–551. 21727007 10.1016/j.tcb.2011.06.002 PMC3164742

[2] Flemming HC, Wingender J (2010) The biofilm matrix. Nature reviews Microbiology 8: 623–633. 20676145 10.1038/nrmicro2415

[3] Branda SS, Vik S, Friedman L, Kolter R (2005) Biofilms: the matrix revisited. Trends in microbiology 13: 20–26. 15639628 10.1016/j.tim.2004.11.006

[4] O'Toole GA (2003) To build a biofilm. Journal of bacteriology 185: 2687–2689. 12700246 10.1128/JB.185.9.2687-2689.2003 PMC154388

[5] Pieters RJ (2011) Carbohydrate mediated bacterial adhesion. Advances in experimental medicine and biology 715: 227–240. 21557067 10.1007/978-94-007-0940-9_14

[6] Patti JM, Hook M (1994) Microbial adhesins recognizing extracellular matrix macromolecules. Current opinion in cell biology 6: 752–758. 7833055 10.1016/0955-0674(94)90104-x

[7] Davies D (2003) Understanding biofilm resistance to antibacterial agents. Nature reviews Drug discovery 2: 114–122. 12563302 10.1038/nrd1008

[8] Walters MC 3rd, Roe F, Bugnicourt A, Franklin MJ, Stewart PS (2003) Contributions of antibiotic penetration, oxygen limitation, and low metabolic activity to tolerance of Pseudomonas aeruginosa biofilms to ciprofloxacin and tobramycin. Antimicrobial agents and chemotherapy 47: 317–323. 12499208 10.1128/AAC.47.1.317-323.2003 PMC148957

[9] Alipour M, Suntres ZE, Omri A (2009) Importance of DNase and alginate lyase for enhancing free and liposome encapsulated aminoglycoside activity against Pseudomonas aeruginosa. The Journal of antimicrobial chemotherapy 64: 317–325. 19465435 10.1093/jac/dkp165

[10] Mah TF, Pitts B, Pellock B, Walker GC, Stewart PS, et al (2003) A genetic basis for Pseudomonas aeruginosa biofilm antibiotic resistance. Nature 426: 306–310. 14628055 10.1038/nature02122

[11] Hou JH, Cohen AE (2012) Motion induced by asymmetric enzymatic degradation of hydrogels. Soft Matter 8: 4616–4624.

[12] Crater JS, Carrier RL (2010) Barrier Properties of Gastrointestinal Mucus to Nanoparticle Transport. Macromolecular Bioscience 10: 1473–1483. 20857389 10.1002/mabi.201000137

[13] Frey S, Gorlich D (2007) A saturated FG-repeat hydrogel can reproduce the permeability properties of nuclear pore complexes. Cell 130: 512–523. 17693259 10.1016/j.cell.2007.06.024

[14] Kirch J, Schneider A, Abou B, Hopf A, Schaefer UF, et al (2012) Optical tweezers reveal relationship between microstructure and nanoparticle penetration of pulmonary mucus. Proceedings of the National Academy of Sciences of the United States of America 109: 18355–18360. 23091027 10.1073/pnas.1214066109 PMC3494950

[15] Schreiber S, Scheid P (1997) Gastric mucus of the guinea pig: proton carrier and diffusion barrier. The American journal of physiology 272: G63–70. 9038877 10.1152/ajpgi.1997.272.1.G63

[16] Stoodley P, Sauer K, Davies DG, Costerton JW (2002) Biofilms as complex differentiated communities. Annual review of microbiology 56: 187–209. 10.1146/annurev.micro.56.012302.160705 12142477

[17] Hentzer M, Teitzel GM, Balzer GJ, Heydorn A, Molin S, et al (2001) Alginate overproduction affects Pseudomonas aeruginosa biofilm structure and function. Journal of bacteriology 183: 5395–5401. 11514525 10.1128/JB.183.18.5395-5401.2001 PMC95424

[18] Colvin KM, Gordon VD, Murakami K, Borlee BR, Wozniak DJ, et al (2011) The pel polysaccharide can serve a structural and protective role in the biofilm matrix of Pseudomonas aeruginosa. PLoS pathogens 7: e1001264. 21298031 10.1371/journal.ppat.1001264 PMC3029257

[19] Costerton JW, Stewart PS, Greenberg EP (1999) Bacterial biofilms: a common cause of persistent infections. Science 284: 1318–1322. 10334980 10.1126/science.284.5418.1318

[20] Stewart PS (2002) Mechanisms of antibiotic resistance in bacterial biofilms. International journal of medical microbiology : IJMM 292: 107–113. 12195733 10.1078/1438-4221-00196

[21] Hogan D, Kolter R (2002) Why are bacteria refractory to antimicrobials? Current opinion in microbiology 5: 472–477. 12354553 10.1016/s1369-5274(02)00357-0

[22] Hoiby N, Bjarnsholt T, Givskov M, Molin S, Ciofu O (2010) Antibiotic resistance of bacterial biofilms. International journal of antimicrobial agents 35: 322–332. 20149602 10.1016/j.ijantimicag.2009.12.011

[23] Church D, Elsayed S, Reid O, Winston B, Lindsay R (2006) Burn wound infections. Clinical microbiology reviews 19: 403–434. 16614255 10.1128/CMR.19.2.403-434.2006 PMC1471990

[24] Kolmos HJ, Thuesen B, Nielsen SV, Lohmann M, Kristoffersen K, et al (1993) Outbreak of infection in a burns unit due to Pseudomonas aeruginosa originating from contaminated tubing used for irrigation of patients. The Journal of hospital infection 24: 11–21. 8101198 10.1016/0195-6701(93)90085-e

[25] Tribou M, Swain G (2010) The use of proactive in-water grooming to improve the performance of ship hull antifouling coatings. Biofouling 26: 47–56. 20390556 10.1080/08927010903290973

[26] Mathieu L, Block JC, Dutang M, Maillard J, Reasoner D (1992) Control of Biofilm Accumulation in Drinking-Water Distribution-Systems. Iwsa Specialized Conference on Quality Aspects of Water Supply 11: 365–376.

[27] Rybtke MT, Jensen PO, Hoiby N, Givskov M, Tolker-Nielsen T, et al (2011) The implication of Pseudomonas aeruginosa biofilms in infections. Inflammation & allergy drug targets 10: 141–157. 21314623 10.2174/187152811794776222

[28] Wozniak DJ, Wyckoff TJ, Starkey M, Keyser R, Azadi P, et al (2003) Alginate is not a significant component of the extracellular polysaccharide matrix of PA14 and PAO1 Pseudomonas aeruginosa biofilms. Proceedings of the National Academy of Sciences of the United States of America 100: 7907–7912. 12810959 10.1073/pnas.1231792100 PMC164686

[29] Byrd MS, Sadovskaya I, Vinogradov E, Lu H, Sprinkle AB, et al (2009) Genetic and biochemical analyses of the Pseudomonas aeruginosa Psl exopolysaccharide reveal overlapping roles for polysaccharide synthesis enzymes in Psl and LPS production. Molecular microbiology 73: 622–638. 19659934 10.1111/j.1365-2958.2009.06795.x PMC4409829

[30] Ma L, Conover M, Lu H, Parsek MR, Bayles K, et al (2009) Assembly and development of the Pseudomonas aeruginosa biofilm matrix. PLoS pathogens 5: e1000354. 19325879 10.1371/journal.ppat.1000354 PMC2654510

[31] Friedman L, Kolter R (2004) Genes involved in matrix formation in Pseudomonas aeruginosa PA14 biofilms. Molecular microbiology 51: 675–690. 14731271 10.1046/j.1365-2958.2003.03877.x

[32] Friedman L, Kolter R (2004) Two genetic loci produce distinct carbohydrate-rich structural components of the Pseudomonas aeruginosa biofilm matrix. Journal of bacteriology 186: 4457–4465. 15231777 10.1128/JB.186.14.4457-4465.2004 PMC438632

[33] Lory S, Merighi M, Hyodo M (2009) Multiple activities of c-di-GMP in Pseudomonas aeruginosa. Nucleic acids symposium series 51–52. 19749255 10.1093/nass/nrp026

[34] Rao J, Damron FH, Basler M, Digiandomenico A, Sherman NE, et al (2011) Comparisons of Two Proteomic Analyses of Non-Mucoid and Mucoid Pseudomonas aeruginosa Clinical Isolates from a Cystic Fibrosis Patient. Frontiers in microbiology 2: 162. 21863142 10.3389/fmicb.2011.00162 PMC3149151

[35] Lee B, Haagensen JA, Ciofu O, Andersen JB, Hoiby N, et al (2005) Heterogeneity of biofilms formed by nonmucoid Pseudomonas aeruginosa isolates from patients with cystic fibrosis. Journal of clinical microbiology 43: 5247–5255. 16207991 10.1128/JCM.43.10.5247-5255.2005 PMC1248443

[36] Tramper-Stranders GA, van der Ent CK, Molin S, Yang L, Hansen SK, et al (2012) Initial Pseudomonas aeruginosa infection in patients with cystic fibrosis: characteristics of eradicated and persistent isolates. Clinical microbiology and infection : the official publication of the European Society of Clinical Microbiology and Infectious Diseases 18: 567–574. 10.1111/j.1469-0691.2011.03627.x 21883670

[37] Wolfgang MC, Kulasekara BR, Liang X, Boyd D, Wu K, et al (2003) Conservation of genome content and virulence determinants among clinical and environmental isolates of Pseudomonas aeruginosa. Proceedings of the National Academy of Sciences of the United States of America 100: 8484–8489. 12815109 10.1073/pnas.0832438100 PMC166255

[38] Colvin KM, Irie Y, Tart CS, Urbano R, Whitney JC, et al (2012) The Pel and Psl polysaccharides provide Pseudomonas aeruginosa structural redundancy within the biofilm matrix. Environmental microbiology 14: 1913–1928. 22176658 10.1111/j.1462-2920.2011.02657.x PMC3840794

[39] Pedersen SS, Espersen F, Hoiby N, Shand GH (1989) Purification, characterization, and immunological cross-reactivity of alginates produced by mucoid Pseudomonas aeruginosa from patients with cystic fibrosis. Journal of clinical microbiology 27: 691–699. 2498389 10.1128/jcm.27.4.691-699.1989 PMC267399

[40] Smith EE, Buckley DG, Wu Z, Saenphimmachak C, Hoffman LR, et al (2006) Genetic adaptation by Pseudomonas aeruginosa to the airways of cystic fibrosis patients. Proceedings of the National Academy of Sciences of the United States of America 103: 8487–8492. 16687478 10.1073/pnas.0602138103 PMC1482519

[41] Evans LR, Linker A (1973) Production and characterization of the slime polysaccharide of Pseudomonas aeruginosa. Journal of bacteriology 116: 915–924. 4200860 10.1128/jb.116.2.915-924.1973 PMC285463

[42] Osman SF, Fett WF, Fishman ML (1986) Exopolysaccharides of the phytopathogen Pseudomonas syringae pv. glycinea. Journal of bacteriology 166: 66–71. 3957873 10.1128/jb.166.1.66-71.1986 PMC214557

[43] Yang L, Hu Y, Liu Y, Zhang J, Ulstrup J, et al (2011) Distinct roles of extracellular polymeric substances in Pseudomonas aeruginosa biofilm development. Environmental microbiology 13: 1705–1717. 21605307 10.1111/j.1462-2920.2011.02503.x

[44] Berlana D, Llop JM, Fort E, Badia MB, Jodar R (2005) Use of colistin in the treatment of multiple-drug-resistant gram-negative infections. American journal of health-system pharmacy : AJHP : official journal of the American Society of Health-System Pharmacists 62: 39–47. 15658071 10.1093/ajhp/62.1.39

[45] Falagas ME, Kasiakou SK (2005) Colistin: the revival of polymyxins for the management of multidrug-resistant gram-negative bacterial infections. Clinical infectious diseases : an official publication of the Infectious Diseases Society of America 40: 1333–1341. 15825037 10.1086/429323

[46] Ceri H, Olson ME, Stremick C, Read RR, Morck D, et al (1999) The Calgary Biofilm Device: new technology for rapid determination of antibiotic susceptibilities of bacterial biofilms. Journal of clinical microbiology 37: 1771–1776. 10325322 10.1128/jcm.37.6.1771-1776.1999 PMC84946

[47] Khan W, Bernier SP, Kuchma SL, Hammond JH, Hasan F, et al (2010) Aminoglycoside resistance of Pseudomonas aeruginosa biofilms modulated by extracellular polysaccharide. International microbiology : the official journal of the Spanish Society for Microbiology 13: 207–212. 21404215 10.2436/20.1501.01.127 PMC3721063

[48] Drusano GL (2007) Pharmacokinetics and pharmacodynamics of antimicrobials. Clinical infectious diseases : an official publication of the Infectious Diseases Society of America 45 (Suppl 1) S89–95. 17582578 10.1086/518137

[49] Kavanaugh NL, Ribbeck K (2012) Selected antimicrobial essential oils eradicate Pseudomonas spp. and Staphylococcus aureus biofilms. Applied and environmental microbiology 78: 4057–4061. 22467497 10.1128/AEM.07499-11 PMC3346404

[50] Zegans ME, Wozniak D, Griffin E, Toutain-Kidd CM, Hammond JH, et al (2012) Pseudomonas aeruginosa exopolysaccharide Psl promotes resistance to the biofilm inhibitor polysorbate 80. Antimicrobial agents and chemotherapy 56: 4112–4122. 22585230 10.1128/AAC.00373-12 PMC3421584

[51] Overhage J, Schemionek M, Webb JS, Rehm BH (2005) Expression of the psl operon in Pseudomonas aeruginosa PAO1 biofilms: PslA performs an essential function in biofilm formation. Applied and environmental microbiology 71: 4407–4413. 16085831 10.1128/AEM.71.8.4407-4413.2005 PMC1183271

[52] Blair DC, Fekety FR Jr, Bruce B, Silva J, Archer G (1975) Therapy of Pseudomonas aeruginosa infections with tobramycin. Antimicrobial agents and chemotherapy 8: 22–29. 809002 10.1128/aac.8.1.22 PMC429254

[53] Lode H (1998) Tobramycin: a review of therapeutic uses and dosing schedules. Current Therapeutic Research 59: 420–453.

[54] Ma L, Lu H, Sprinkle A, Parsek MR, Wozniak DJ (2007) Pseudomonas aeruginosa Psl is a galactose- and mannose-rich exopolysaccharide. Journal of bacteriology 189: 8353–8356. 17631634 10.1128/JB.00620-07 PMC2168683

[55] Decho AW, Visscher PT, Reid RP (2005) Production and cycling of natural microbial exopolymers (EPS) within a marine stromatolite. Palaeogeography Palaeoclimatology Palaeoecology 219: 71–86.

[56] Hammond AA, Miller KG, Kruczek CJ, Dertien J, Colmer-Hamood JA, et al (2011) An in vitro biofilm model to examine the effect of antibiotic ointments on biofilms produced by burn wound bacterial isolates. Burns : journal of the International Society for Burn Injuries 37: 312–321. 21130579 10.1016/j.burns.2010.09.017 PMC3034806

[57] Frank DN, Wysocki A, Specht-Glick DD, Rooney A, Feldman RA, et al (2009) Microbial diversity in chronic open wounds. Wound repair and regeneration : official publication of the Wound Healing Society [and] the European Tissue Repair Society 17: 163–172. 10.1111/j.1524-475X.2009.00472.x 19320883

[58] Bowler PG, Duerden BI, Armstrong DG (2001) Wound microbiology and associated approaches to wound management. Clinical microbiology reviews 14: 244–269. 11292638 10.1128/CMR.14.2.244-269.2001 PMC88973

[59] Fazli M, Bjarnsholt T, Kirketerp-Moller K, Jorgensen B, Andersen AS, et al (2009) Nonrandom distribution of Pseudomonas aeruginosa and Staphylococcus aureus in chronic wounds. Journal of clinical microbiology 47: 4084–4089. 19812273 10.1128/JCM.01395-09 PMC2786634

[60] Rogers GB, Carroll MP, Serisier DJ, Hockey PM, Jones G, et al (2004) characterization of bacterial community diversity in cystic fibrosis lung infections by use of 16s ribosomal DNA terminal restriction fragment length polymorphism profiling. Journal of clinical microbiology 42: 5176–5183. 15528712 10.1128/JCM.42.11.5176-5183.2004 PMC525137

[61] Kooistra-Smid M, Nieuwenhuis M, van Belkum A, Verbrugh H (2009) The role of nasal carriage in Staphylococcus aureus burn wound colonization. FEMS immunology and medical microbiology 57: 1–13. 19486150 10.1111/j.1574-695X.2009.00565.x

[62] Busch NA, Zanzot EM, Loiselle PM, Carter EA, Allaire JE, et al (2000) A model of infected burn wounds using Escherichia coli O18:K1:H7 for the study of gram-negative bacteremia and sepsis. Infection and immunity 68: 3349–3351. 10816484 10.1128/iai.68.6.3349-3351.2000 PMC97598

[63] Revathi G, Puri J, Jain BK (1998) Bacteriology of burns. Burns : journal of the International Society for Burn Injuries 24: 347–349. 9688200 10.1016/s0305-4179(98)00009-6

[64] Percival SL, Thomas J, Linton S, Okel T, Corum L, et al (2012) The antimicrobial efficacy of silver on antibiotic-resistant bacteria isolated from burn wounds. International wound journal 9: 488–493. 22182219 10.1111/j.1742-481X.2011.00903.x PMC7950351

[65] Mah TF, O'Toole GA (2001) Mechanisms of biofilm resistance to antimicrobial agents. Trends in microbiology 9: 34–39. 11166241 10.1016/s0966-842x(00)01913-2

[66] Nichols WW, Dorrington SM, Slack MP, Walmsley HL (1988) Inhibition of tobramycin diffusion by binding to alginate. Antimicrobial agents and chemotherapy 32: 518–523. 3132093 10.1128/aac.32.4.518 PMC172213

[67] Hatch RA, Schiller NL (1998) Alginate lyase promotes diffusion of aminoglycosides through the extracellular polysaccharide of mucoid Pseudomonas aeruginosa. Antimicrobial agents and chemotherapy 42: 974–977. 9559826 10.1128/aac.42.4.974 PMC105585

[68] Sadovskaya I, Vinogradov E, Li J, Hachani A, Kowalska K, et al (2010) High-level antibiotic resistance in Pseudomonas aeruginosa biofilm: the ndvB gene is involved in the production of highly glycerol-phosphorylated beta-(1→3)-glucans, which bind aminoglycosides. Glycobiology 20: 895–904. 20348539 10.1093/glycob/cwq047

[69] Al-Bakri AG, Gilbert P, Allison DG (2005) Influence of gentamicin and tobramycin on binary biofilm formation by co-cultures of Burkholderia cepacia and Pseudomonas aeruginosa. Journal of basic microbiology 45: 392–396. 16187262 10.1002/jobm.200510011

[70] Burmolle M, Webb JS, Rao D, Hansen LH, Sorensen SJ, et al (2006) Enhanced biofilm formation and increased resistance to antimicrobial agents and bacterial invasion are caused by synergistic interactions in multispecies biofilms. Applied and environmental microbiology 72: 3916–3923. 16751497 10.1128/AEM.03022-05 PMC1489630

[71] Hoffman LR, Deziel E, D'Argenio DA, Lepine F, Emerson J, et al (2006) Selection for Staphylococcus aureus small-colony variants due to growth in the presence of Pseudomonas aeruginosa. Proceedings of the National Academy of Sciences of the United States of America 103: 19890–19895. 17172450 10.1073/pnas.0606756104 PMC1750898

[72] Kara D, Luppens SBI, Cate JM (2006) Differences between single- and dual-species biofilms of Streptococcus mutans and Veillonella parvula in growth, acidogenicity and susceptibility to chlorhexidine. European Journal of Oral Sciences 114: 58–63. 16460342 10.1111/j.1600-0722.2006.00262.x

[73] Lagendijk EL, Validov S, Lamers GE, de Weert S, Bloemberg GV (2010) Genetic tools for tagging Gram-negative bacteria with mCherry for visualization in vitro and in natural habitats, biofilm and pathogenicity studies. FEMS microbiology letters 305: 81–90. 20180857 10.1111/j.1574-6968.2010.01916.x

[74] Sambrook J, Russell DW (2001) Molecular cloning : a laboratory manual. Cold Spring Harbor, N.Y.: Cold Spring Harbor Laboratory Press.

[75] Merritt JH, Kadouri DE, O'Toole GA (2011) Growing and analyzing static biofilms. Current protocols in microbiology. Current Protocols in Microbiology 22: 1B.1.1–1B.1.18. 10.1002/9780471729259.mc01b01s00 PMC4568995 18770545

[76] Schneider CA, Rasband WS, Eliceiri KW (2012) NIH Image to ImageJ: 25 years of image analysis. Nature methods 9: 671–675. 22930834 10.1038/nmeth.2089 PMC5554542

