# Supplementary figures and images for: The Extracellular Matrix Component Psl Provides Fast-Acting Antibiotic Defense in Pseudomonas aeruginosa Biofilms
Source: PLoS Pathog. 2013 Aug 8;9(8):e1003526. doi: 10.1371/journal.ppat.1003526 (PMC3738486; doi:10.1371/journal.ppat.1003526)

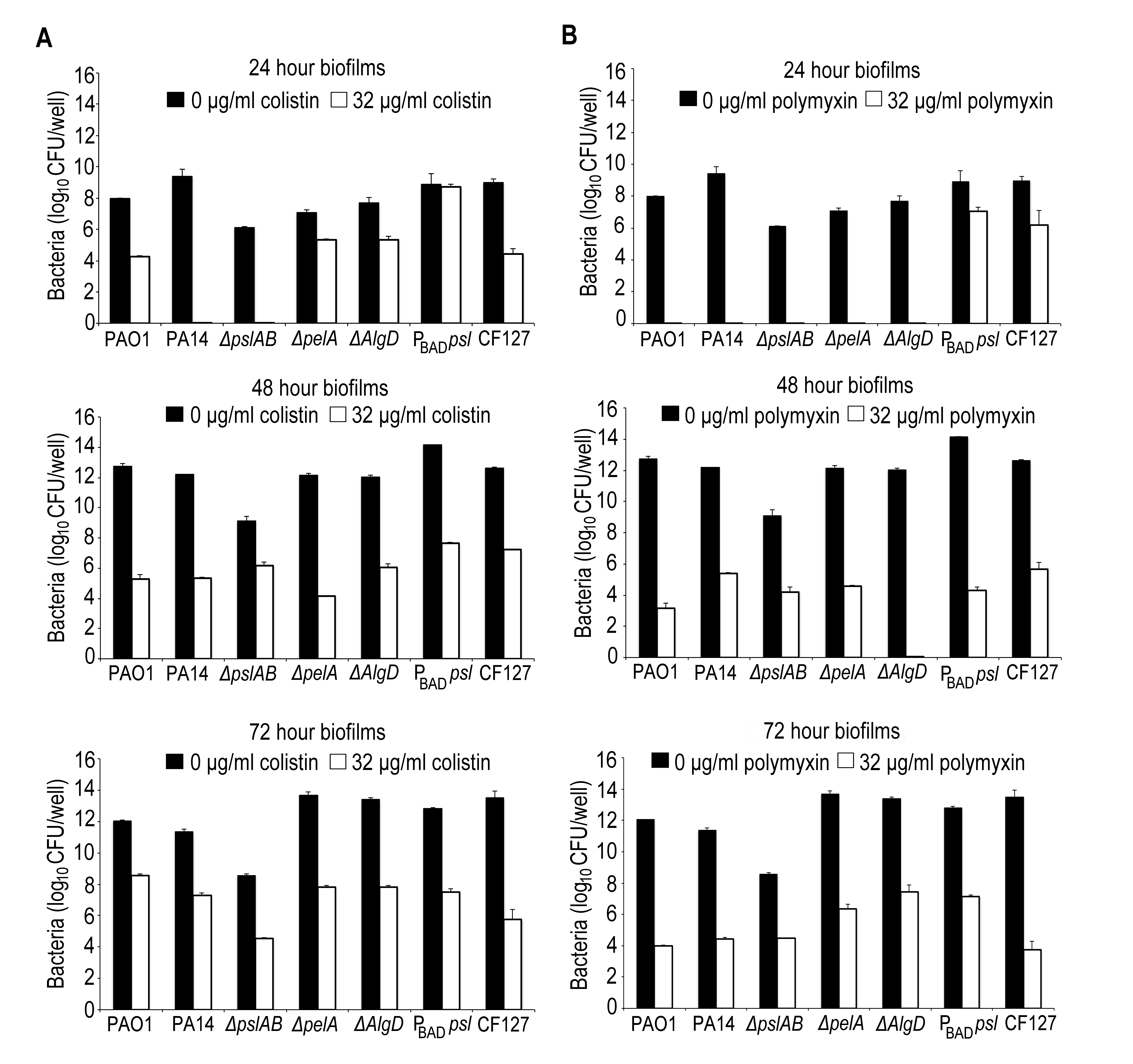

Supplement: Figure S1 — Psl protects 24-hour biofilms from cationic antimicrobial peptides. WT PAO1, WT PA14, ΔpslAB, ΔpelA, ΔalgD, P BAD- psl, and CF127 biofilms were grown for 24, 48, 72 hours in microtiter plates. After a 2-hour treatment with 32 µg/ml of colistin (A) or polymyxin B (B), cell viability was measure and reported at CFU (log10). Psl-mediated protection was apparent for 24-hour biofilms, but dispensable at later time points. (TIF) [file ppat.1003526.s001.tif]

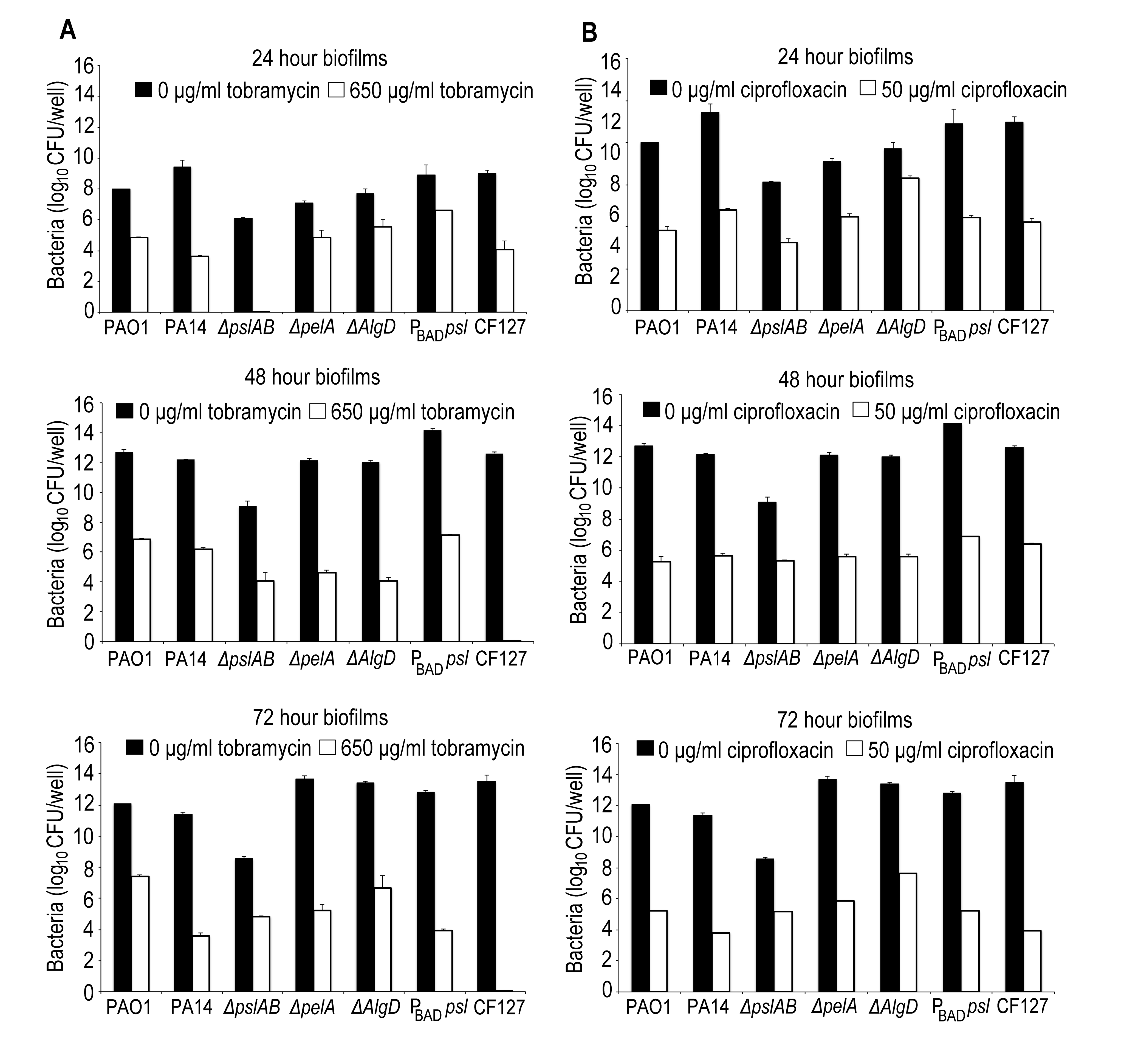

Supplement: Figure S2 — Psl protects 24-hour biofilms from cationic antimicrobial peptides. WT PAO1, WT PA14, ΔpslAB, ΔpelA, ΔalgD, P BAD- psl, and CF127 biofilms were grown for 24, 48, 72 hours in microtiter plates. After a 2-hour treatment with 650 µg/ml of tobramycin (A) or 50 µg/ml ciprofloxacin (B), cell viability was measure and reported at CFU (log10). Psl-mediated protection was critical for ΔpslAB 24-hour biofilms treated with tobramycin, but was not required at 48 or 72 hours. Tolerance to ciprofloxacin in strains lacking Psl was not as apparent with this assay. (TIF) [file ppat.1003526.s002.tif]

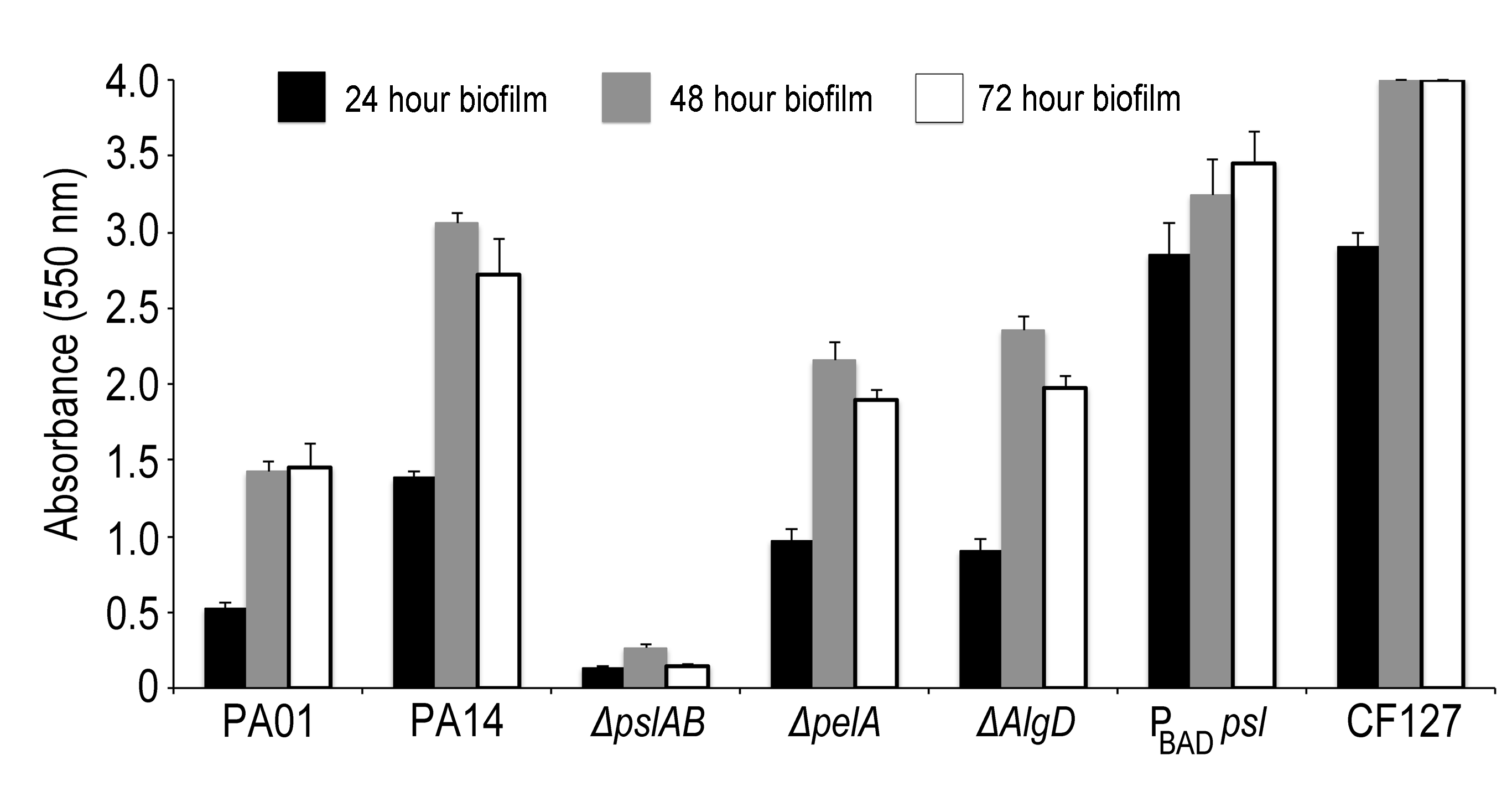

Supplement: Figure S3 — A deletion of pslAB in the WT PAO1 background reduces total biomass in an in vitro biofilm model. Crystal violet assays were used to quantify the total biomass for air-liquid interface biofilms grown in 96 well plates for 24, 48, and 72-hour biofilms. The total biomass for ΔpslAB was reduced relative to WT PAO1 for all time points measured. Although PA14 does not produce Psl, a reduction in biomass was not observed, presumably due to other contributing polymers in the biofilm matrix. Deletions in pelA or ΔalgD did not reduce the total biomass relative to WT PAO1. Both P BAD- psl and CF127 had a greater than 2-fold increase in biomass for each time point compared to WT PAO1. (TIF) [file ppat.1003526.s003.tif]

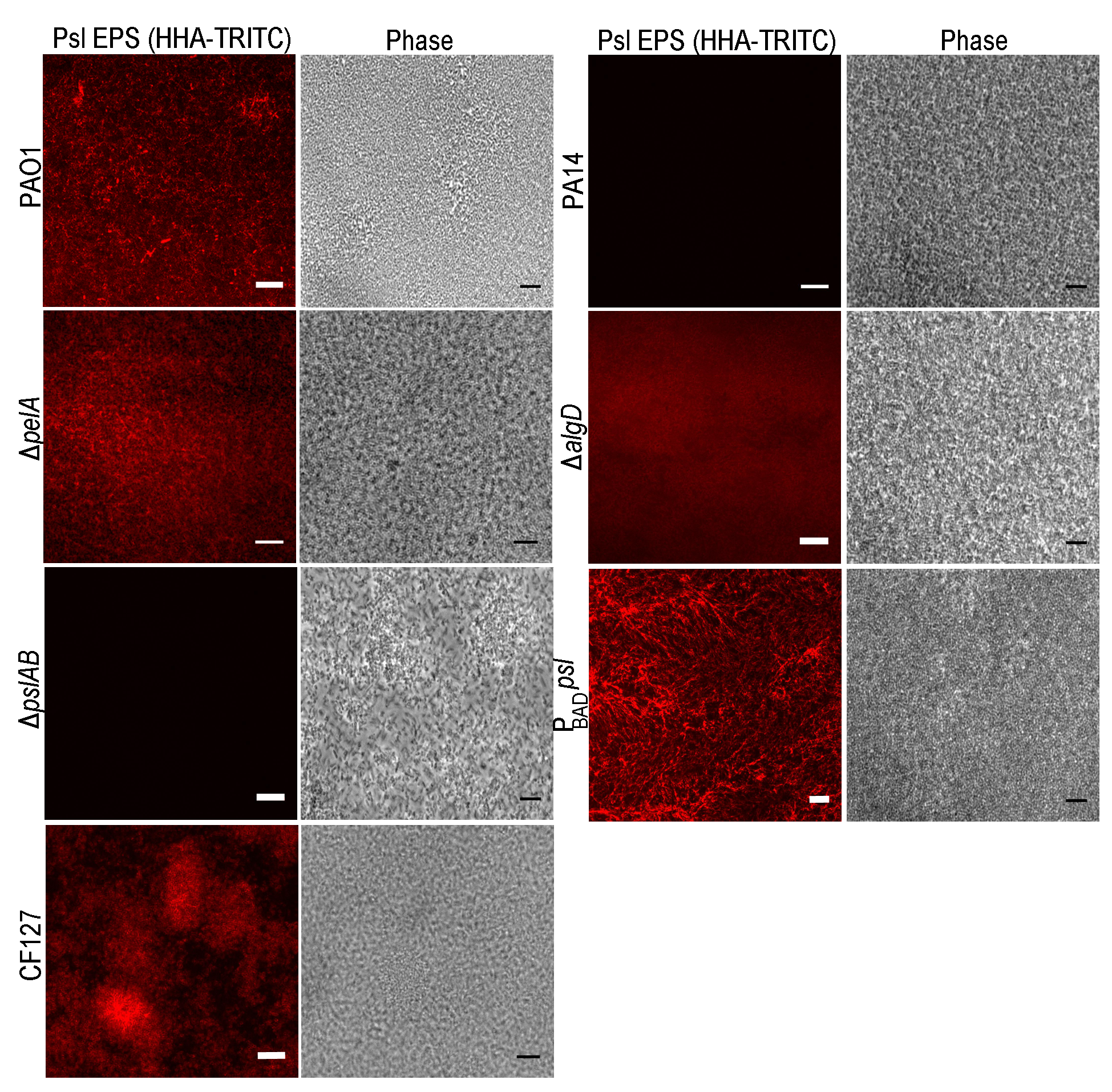

Supplement: Figure S4 — Lectin staining reveals patterns of Psl distribution in 24-hour biofilms. Fluorescently labeled HHA stained Psl in WT PAO1, ΔpelA, ΔalgD, P BAD- psl, and CF127 biofilms [54]. Both PA14 and ΔpslAB lack Psl in the matrix and did not bind HHA. Psl was distributed as a localized, fibrous material associated with WT PAO1 and P BAD- psl biofilms, while in ΔpelA and ΔalgD, the Psl matrix was uniformly distributed throughout the biofilm. HHA localized to microcolonies in CF127 biofilms indicating that Psl was enriched in these structures. Scale bars represent 10 µm. (TIF) [file ppat.1003526.s004.tif]

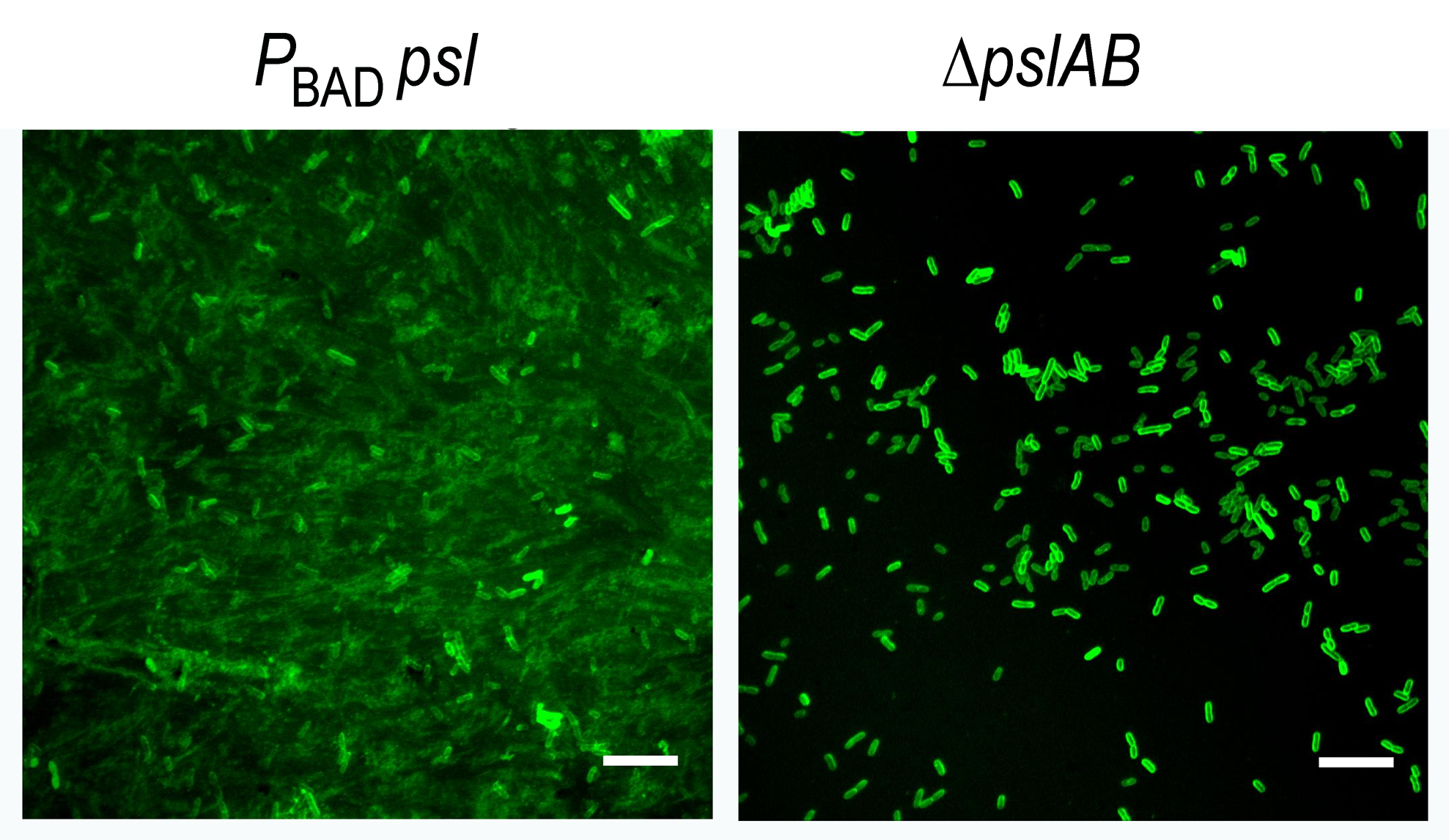

Supplement: Figure S5 — Polymyxin B interaction with the extracellular matrix in planktonic cells. Images of over-producing Psl (P BAD- psl) and Psl deficient (ΔpslAB) cells after a 2-hour challenge with fluorescent polymyxin B. Polymyxin B accumulates in the EPS of Psl over-expressing cells, but appears to bind directly to the cell surface in the Psl deletion strain. Scale bars represent 10 µm. (TIF) [file ppat.1003526.s005.tif]

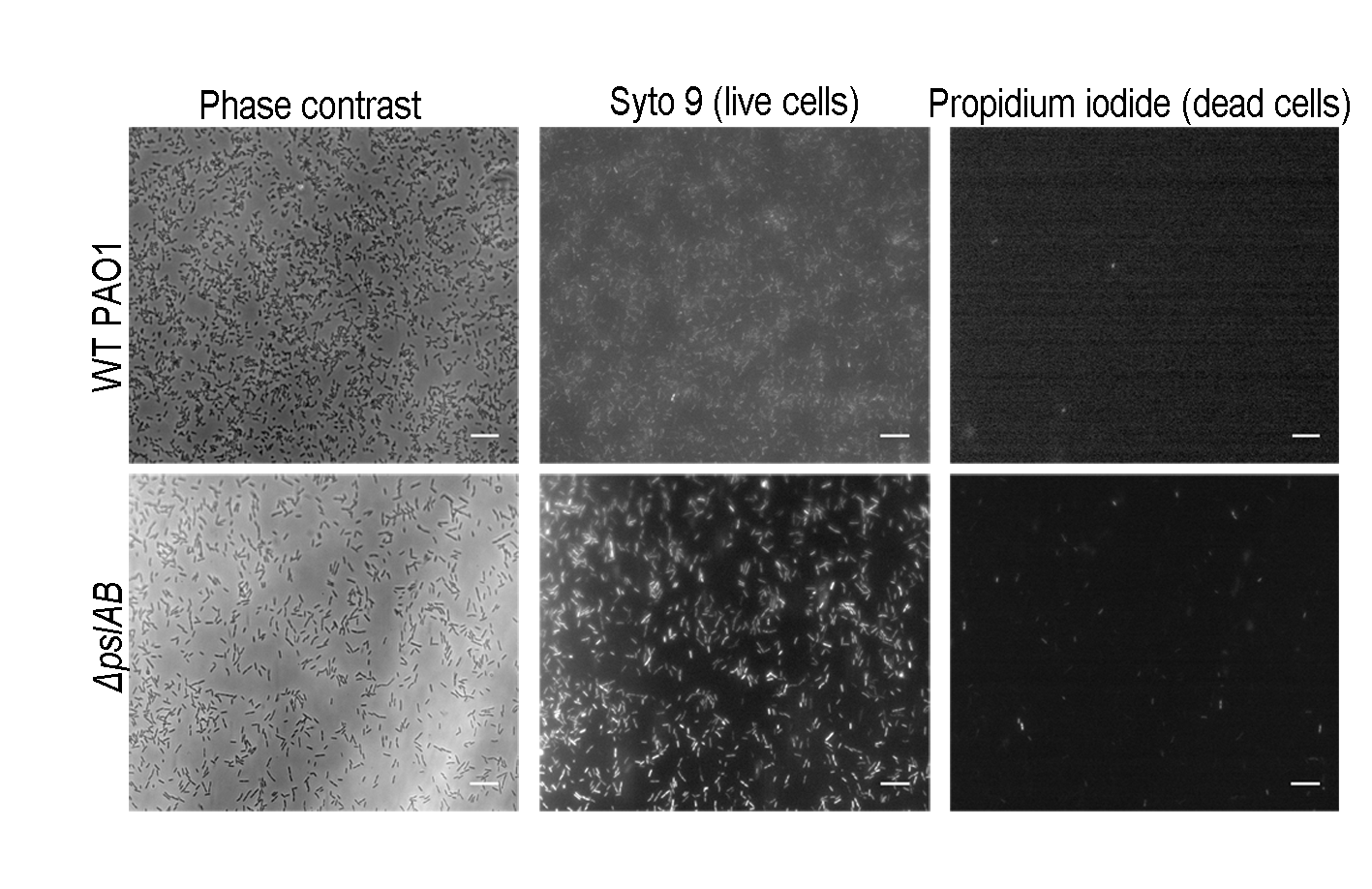

Supplement: Figure S6 — Biofilms treated with water only did not contribute to cell death in microfluidic channel. To serve as a control, biofilms grown in microfluidic channels were treated with sterile water only and monitored for cell death for 2 hours. Images of WT PAO1 and mutant strain ΔpslAB stained with Syto 9 (live cells) and propidium iodide (dead cells) were acquired and compared after 1 hour of treatment with water only. (TIF) [file ppat.1003526.s006.tif]

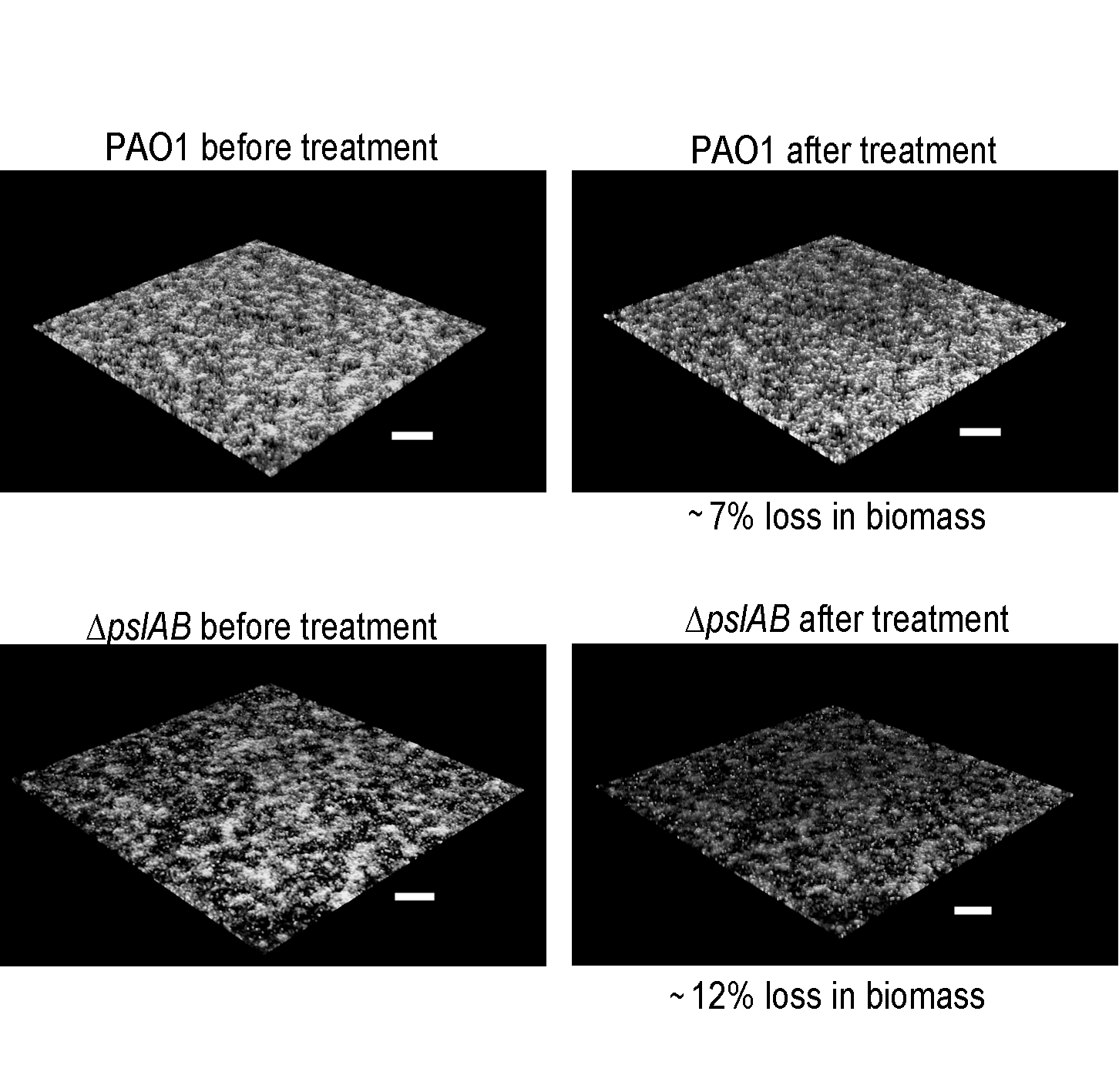

Supplement: Figure S7 — Biomass before and after treatment with colistin. 3-D projection of confocal images of WT PAO1 and mutant strain ΔpslAB were acquired and compared before (at 24 hours) and after treatment (at 26 hours) with 20 µg/ml colistin. Cells were stained with Syto9 (Molecular Probes) and counted in the series of xyz images. Scale bars represent 25 µm. (TIF) [file ppat.1003526.s007.tif]

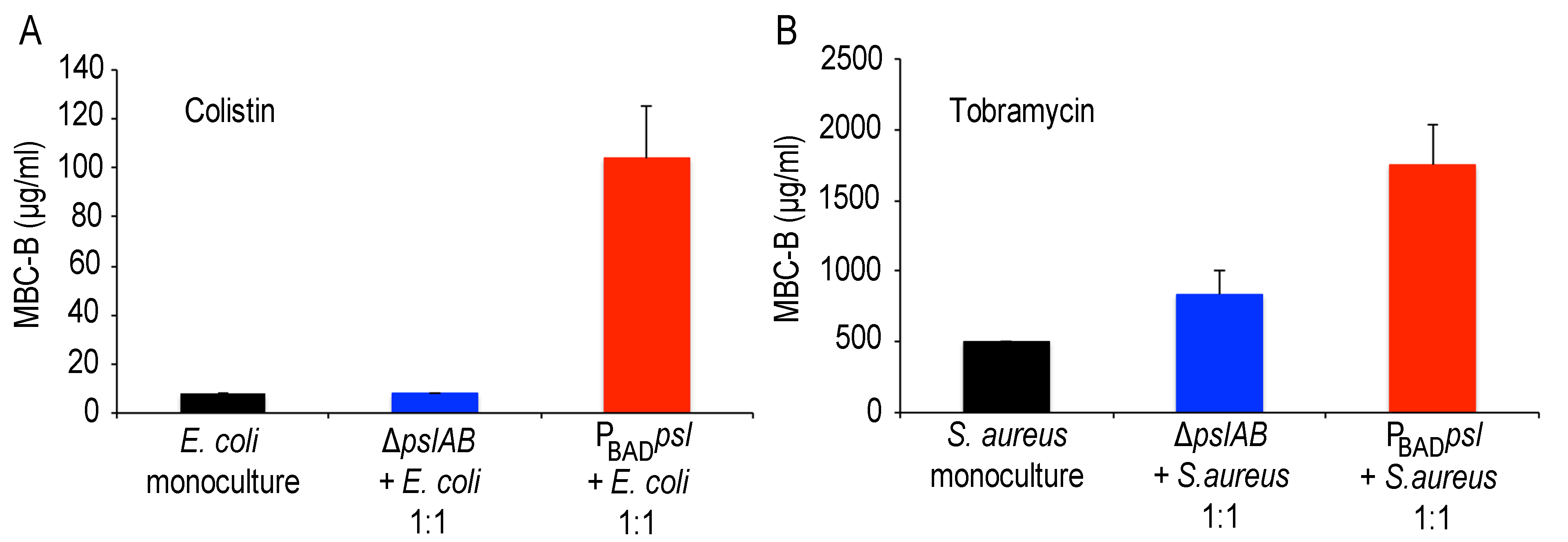

Supplement: Figure S8 — Psl increases MBC-B for E. coli and S. aureus. MBC-B assay reveals an increase in tolerance toward colistin for E. coli and P BAD- psl biofilms (A). Tolerance is also observed toward tobramycin for S. aureus and P BAD- psl biofilms, but to a lesser extent (B). (TIF) [file ppat.1003526.s008.tif]
