# Supplementary material for: The Extracellular Matrix Component Psl Provides Fast-Acting Antibiotic Defense in Pseudomonas aeruginosa Biofilms
Source: PLoS Pathog. 2013 Aug 8;9(8):e1003526. doi: 10.1371/journal.ppat.1003526 (PMC3738486; doi:10.1371/journal.ppat.1003526)
Supplement: Text S1 — Description of the strains and antibiotics used in this study. (DOCX) [file ppat.1003526.s009.docx]

| Strain | Description | Reference |
| --- | --- | --- |
| PAO1 | *P. aeruginosa* wild-type laboratory strain | [[1](#_ENREF_1)] |
| PAO1∆pslAB | Non-polar disruption of pslAB in PAO1 | [[2](#_ENREF_2)] |
| PAO1∆algD | *algD*::*tet* in PAO1 | [[3](#_ENREF_3)] |
| PAO1∆pelA | markerless deletion of *pelA* in PAO1 | [[4](#_ENREF_4)] |
| PAO1-P_BAD_*psl* | Chromosomal psl regulated by inducible araC-pBAD | [[5](#_ENREF_5)] |
| PA14 | *P. aeruginosa* wild-type laboratory strain | [[6](#_ENREF_6)] |
| CF127 | *P. aeruginosa* cystic fibrosis isolate | [[7](#_ENREF_7)] |
| EMG2 | *E. coli* K12 wild-type laboratory strain | [[8](#_ENREF_8)] |
| UAMS-1 | *S. aureus* osteomyelitis isolate | [[9](#_ENREF_9)] |

**Table S1.** Strains used in this study.

**Table S2.** Antibiotics used in this study.

| Antibiotic name | Formula | Molecular weight (g/mol) | Net charge | References |
| --- | --- | --- | --- | --- |
| Colistin (Polymyxin E) | C_53_H_102_N_16_O_17_S | 1267.6 | positive | [[10](#_ENREF_10)] |
| Polymyxin B | C_56_H_100_N_16_O_17_S | 1301.56 | positive | [[10](#_ENREF_10)] |
| Tobramycin | C_18_H_37_N_5_O_9_ | 467.51 | positive | [[11](#_ENREF_11)] |
| Ciprofloxacin | C_17_H_18_FN_3_O_3_ | 331.346 | negative | [[12](#_ENREF_12)] |

**Supporting References**

1. Holloway BW (1955) Genetic recombination in Pseudomonas aeruginosa. Journal of general microbiology 13: 572-581.

2. Jackson KD, Starkey M, Kremer S, Parsek MR, Wozniak DJ (2004) Identification of psl, a locus encoding a potential exopolysaccharide that is essential for Pseudomonas aeruginosa PAO1 biofilm formation. Journal of bacteriology 186: 4466-4475.

3. Whitchurch CB, Erova TE, Emery JA, Sargent JL, Harris JM, et al. (2002) Phosphorylation of the Pseudomonas aeruginosa response regulator AlgR is essential for type IV fimbria-mediated twitching motility. Journal of bacteriology 184: 4544-4554.

4. Borlee BR, Goldman AD, Murakami K, Samudrala R, Wozniak DJ, et al. (2010) Pseudomonas aeruginosa uses a cyclic-di-GMP-regulated adhesin to reinforce the biofilm extracellular matrix. Molecular microbiology 75: 827-842.

5. Ma L, Jackson KD, Landry RM, Parsek MR, Wozniak DJ (2006) Analysis of Pseudomonas aeruginosa conditional psl variants reveals roles for the psl polysaccharide in adhesion and maintaining biofilm structure postattachment. Journal of bacteriology 188: 8213-8221.

6. Rahme LG, Stevens EJ, Wolfort SF, Shao J, Tompkins RG, et al. (1995) Common virulence factors for bacterial pathogenicity in plants and animals. Science 268: 1899-1902.

7. Wolfgang MC, Kulasekara BR, Liang X, Boyd D, Wu K, et al. (2003) Conservation of genome content and virulence determinants among clinical and environmental isolates of Pseudomonas aeruginosa. Proceedings of the National Academy of Sciences of the United States of America 100: 8484-8489.

8. Bachmann BJ (1972) Pedigrees of some mutant strains of Escherichia coli K-12. Bacteriological reviews 36: 525-557.

9. Gillaspy AF, Hickmon SG, Skinner RA, Thomas JR, Nelson CL, et al. (1995) Role of the accessory gene regulator (agr) in pathogenesis of staphylococcal osteomyelitis. Infection and immunity 63: 3373-3380.

10. Vaara M, Fox J, Loidl G, Siikanen O, Apajalahti J, et al. (2008) Novel polymyxin derivatives carrying only three positive charges are effective antibacterial agents. Antimicrobial agents and chemotherapy 52: 3229-3236.

11. Josephson L, Houle P, Haggerty M (1979) Stability of dilute solutions of gentamicin and tobramycin. Clinical chemistry 25: 298-300.

12. Ramchandani M, Robinson D (1998) In vitro and in vivo release of ciprofloxacin from PLGA 50:50 implants. Journal of controlled release : official journal of the Controlled Release Society 54: 167-175.
